# Targeting SARS-CoV-2 Structural and Accessory Proteins: Emerging Opportunities for Small-Molecule Coronavirus Antivirals

**DOI:** 10.3390/pharmaceutics18060706

**Published:** 2026-06-08

**Authors:** Exequiel O. J. Porta, Dana F. AlKharboush, Lauren Jackson, Felix Pang, Aylin Darin, Joy Louka, Xinyue Shi, Geoffrey Wells, Frank Kozielski

**Affiliations:** 1 UCL School of Pharmacy, University College London, London WC1N 1AX, UK; 2 Department of Pharmaceutical Chemistry, Faculty of Pharmacy, King Abdulaziz University, Jeddah 22252, Saudi Arabia

**Keywords:** SARS-CoV-2, direct-acting antivirals, structural proteins, accessory proteins, Spike protein, membrane protein, envelope protein, nucleocapsid protein, ORF3a, antiviral resistance, pan-coronavirus preparedness

## Abstract

Although antiviral development against severe acute respiratory syndrome coronavirus 2 (SARS-CoV-2) has been dominated by replication-directed strategies, structural and accessory proteins offer a complementary and increasingly important opportunity for small-molecule intervention. These proteins control key processes outside the core replication machinery, including viral entry, membrane remodelling, virion assembly, egress, and host immune modulation, thereby expanding the mechanistic scope of antiviral design. However, many of these targets are membrane-associated, oligomeric, conformationally dynamic, or function through protein–protein interactions, creating distinct challenges in target validation, assay design, and chemical optimisation. In this review, we comprehensively and critically evaluate the structural and accessory proteomes of SARS-CoV-2, with a strict focus on small-molecule tractability and translational relevance. We highlight the most credible direct-acting opportunities, focusing on the membrane (M), envelope (E), and nucleocapsid (N) structural proteins, together with the accessory protein open reading frame 3a (ORF3a), for which emerging chemical matter strengthens confidence in druggability. In contrast, Spike (S) and several host-interface accessory proteins, including ORF6, ORF8, ORF9b, and ORF10, are best viewed as more selective or earlier-stage opportunities that require stronger on-target chemical validation. Emphasis is placed on structural accessibility, mechanism-based assay systems, evidence quality, cellular and in vivo activity, and developability constraints relevant to exposure at the infection site. Rather than replacing replication-directed antivirals, these non-canonical targets are best considered adjunctive or complementary components of future combination strategies designed to broaden antiviral coverage, enhance robustness, and improve pandemic preparedness.

## 1. Introduction

**Figure 1:**
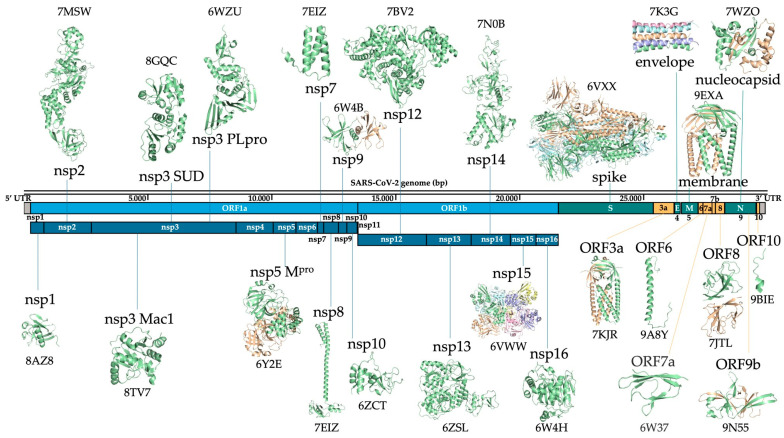
Genome organisation of SARS-CoV-2. The ~30 kb positive-sense RNA genome encodes non-structural proteins (Nsps) within ORF1a/ORF1b and, in the 3′ region, the structural (S, E, M, N) and accessory proteins [34]. Structural and accessory proteins are expressed from a 3′-nested set of subgenomic mRNAs synthesised by discontinuous transcription. Representative available protein structures are shown with their Protein Data Bank (PDB) accession codes. Where experimentally determined and biologically relevant, proteins are represented in their functional oligomeric state, including dimers, trimers, pentamers, or hexamers. Monomeric proteins or isolated domains are shown where the monomeric form is biologically relevant, where the corresponding oligomeric assembly is not available or not structurally resolved, or where a domain-level structure is most informative. Monomers are shown in green; dimers in green and beige; the Spike trimer in green, beige, and cyan; the Envelope pentamer in green, beige, cyan, purple, and pink; and the Nsp15 hexamer in green, beige, cyan, purple, pink, and yellow.

Since its emergence in 2019, severe acute respiratory syndrome coronavirus 2 (SARS-CoV-2) has underscored the need for antiviral strategies that remain effective despite viral evolution, incomplete immune protection, and the practical limitations of first-generation therapies [1,2,3,4,5,6]. While the most clinically successful direct-acting antivirals (DAAs) have targeted enzymes within the non-structural proteome, the broader coronavirus life cycle contains additional vulnerabilities that may enable mechanistically complementary intervention [7,8,9]. In particular, the structural and accessory proteins of SARS-CoV-2 govern a distinct layer of viral biology that extends beyond genome replication (Figure 1), encompassing host–cell entry, membrane remodelling, virion assembly, genome packaging, egress, and host-pathway subversion [10,11,12]. These processes shape viral fitness, transmissibility, and pathogenesis and therefore define a complementary target space for next-generation antiviral design. A detailed discussion of non-structural proteins (Nsps) as antiviral targets is beyond the scope of the present review and is addressed separately in the companion article focused on the SARS-CoV-2 non-structural proteome [13].

The four structural proteins, Spike (S), Envelope (E), Membrane (M), and Nucleocapsid (N), form the physical virion, but each also contributes active and potentially druggable functions [14]. Spike mediates receptor engagement and membrane fusion and thus remains the principal determinant of host–cell entry [15]. By contrast, E and M coordinate envelope organisation, secretory-pathway remodelling, and virion assembly, whereas N controls genome encapsidation, ribonucleoprotein organisation, and assembly-linked protein—ribonucleic acid (RNA) interactions [16]. Collectively, these proteins offer opportunities to disrupt entry, fusion, assembly, and release through mechanisms that are orthogonal to inhibition of the replication–transcription complex [17]. However, they also pose a significant drug-discovery challenge: many are membrane-associated, oligomeric, conformationally dynamic, or dominated by protein–protein and protein–RNA interfaces rather than deep catalytic pockets. This has historically made small-molecule development more challenging, but it also creates opportunities for conformation-selective, allosteric, or interface-disrupting intervention strategies that may complement, rather than replace, enzyme-directed therapies [18].

The accessory proteome adds another layer of therapeutic interest. Although accessory proteins are generally dispensable for efficient replication in standard cell cultures, they play disproportionate roles in immune evasion, inflammatory dysregulation, tissue adaptation, and virulence in more physiologically relevant settings [19,20]. Several accessory proteins act at host-interface nodes that are mechanistically distinct from canonical replication enzymes. Mechanistic anchors include open reading frame 6 (ORF6), which binds the nucleoporin 98 (Nup98)–ribonucleic acid export 1 (Rae1) nuclear pore complex to block host messenger RNA (mRNA) export and signal transducer and activator of transcription 1 (STAT1) nuclear import, thereby crippling interferon (IFN) signalling; ORF9b, which targets translocase of outer mitochondrial membrane 70 (TOM70) to blunt retinoic acid-inducible gene I (RIG-I)/mitochondrial antiviral-signalling protein (MAVS) signalling; ORF3a, a viroporin reported to promote NOD-, LRR- and pyrin domain-containing protein 3 (NLRP3) inflammasome activation and cell stress; and ORF7a, which antagonises bone marrow stromal antigen 2 (BST-2), also known as tetherin or cluster of differentiation 317 (CD317), facilitating virion release [21,22,23,24,25,26,27,28]. ORF8 exemplifies their contribution to pathogenesis: it downregulates major histocompatibility complex class I (MHC-I) to evade cytotoxic T cells, and naturally occurring Δ382 deletions, which remove ORF8, were associated with milder disease early in the pandemic, highlighting both immune-evasion function and the variability of accessory loci [29,30]. In contrast, ORF10 remains debated: multiple studies find it non-essential and dispensable for replication in vitro and in vivo, although overexpression studies have suggested possible innate-immunity effects; overall, its druggability and clinical relevance are uncertain [31,32].

These features make accessory proteins particularly attractive adjunctive targets. Inhibiting them is unlikely to sterilise the infection in isolation, but it may reduce pathogenesis, blunt viral immune evasion, and enhance the performance of DAAs that suppress replication more proximally. Their chief liabilities, however, include higher sequence variability, context-dependent phenotypes, and greater dependence on physiologically relevant assay systems to establish on-target activity.

Several reviews have previously summarised the biology, structure, pathogenesis and therapeutic relevance of SARS-CoV-2 structural and accessory proteins [18,19,20,33]. However, the present review differs in both scope and purpose. Earlier reviews have mainly focused on viral protein biology, immune evasion, host–pathogen interactions, structural features, or broad therapeutic relevance. In contrast, this review is framed from a medicinal chemistry and translational drug-discovery perspective, with a strict focus on small-molecule tractability. We evaluate each structural and selected accessory protein according to ligandability, assay readiness, quality of available chemical matter, cellular and in vivo evidence, target-engagement confidence, resistance considerations, pharmacokinetics/pharmacodynamics (PK/PD) or delivery constraints, and current development status. This approach allows us to distinguish targets with credible direct-acting small-molecule potential, such as M, from targets that remain early-stage, adjunctive, host-interface, or virulence-modifying opportunities.

Accordingly, the structural and accessory proteomes should not be viewed as substitutes for clinically validated non-structural enzyme targets, but as a complementary and more selective layer of antiviral opportunity. Their greatest strategic value lies in expanding the mechanistic diversity of the antiviral toolkit through entry, fusion, assembly, egress, and immune-evasion pathways. Rather than treating all such proteins as equivalent targets, this review aims to define where the non-canonical antiviral landscape is already actionable, where it remains exploratory, and how these targets may contribute to more resilient coronavirus therapeutic strategies in the future.

## 2. Structural Proteins as Molecular Targets

**Figure 2:**
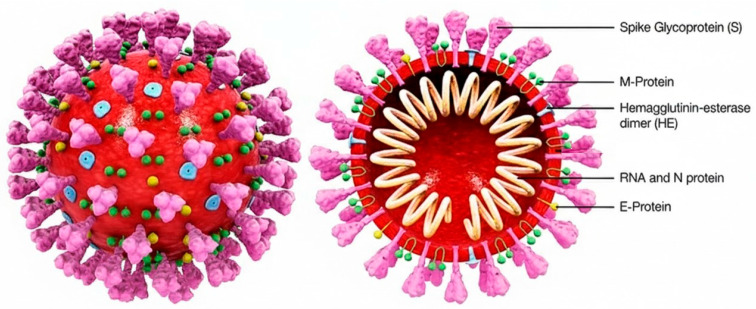
Coronavirus virion architecture. Surface (**left**) and cut-away (**right**) views showing Spike (S) trimers, membrane (M) protein, envelope (E) protein, and the ribonucleoprotein core (genomic RNA wrapped by N). Hemagglutinin-esterase (HE) is present in some β-coronaviruses (lineage A) but is not encoded by SARS-CoV-2.

SARS-CoV-2 comprises four structural proteins: S, E, M, and N (Figure 2). These proteins not only form the physical virion but also play a role in pathogenic processes [35]. Antivirals targeting these structural proteins primarily aim to disrupt viral entry (S) or influence viral assembly and release (E, M, N). Although small-molecule development against structural proteins has been less successful than that against Nsps, notable progress has been made for M. Below, we examine each structural protein as a potential drug target.

### 2.1. Spike (S) Glycoprotein

#### 2.1.1. Biology and Rationale

**Figure 3:**
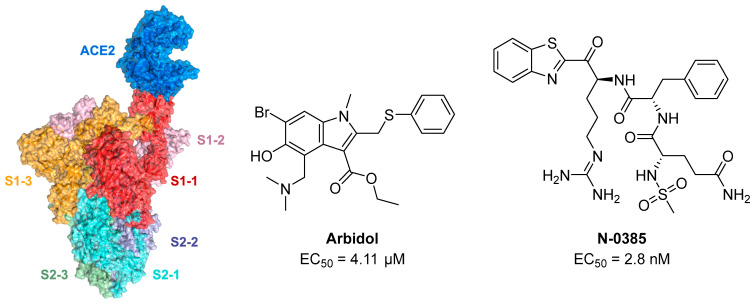
(**Left**): Cryo-EM structure of the SARS-CoV-2 spike trimer in complex with one ACE2 molecule (PDB ID 7A94). The S1 subdomains (Q14-R685) are shown in red, pink, and orange, the S2 subdomains (S686-D1146) in cyan, purple, and green, and ACE2 in blue. Bound N-acetylg lucosamine and zinc ions are not included in the figure. (**Right**): Arbidol (Umifenovir) [78], a reported spike-fusion/trimer-stabilising inhibitor, and N-0385 [62], a nM TMPRSS2 inhibitor that blocks spike priming.

The S glycoprotein (1273 amino acids) is the principal determinant of SARS-CoV-2 entry, mediating receptor engagement and membrane fusion. S is a trimeric class I fusion protein composed of S1 (receptor binding) and S2 (fusion), and it initiates infection by binding to angiotensin-converting enzyme 2 (ACE2; Figure 3), followed by proteolytic activation and large conformational rearrangements that drive membrane merger [36,37]. Because S is surface-exposed and essential for entry, it is an attractive antiviral target. In practice, however, the most clinically advanced S-directed modalities have been biologics such as vaccines, neutralising monoclonal antibodies (mAbs), and related protein-based formats, reflecting both the accessibility of the S ectodomain and the historical difficulty of achieving potent, selective small-molecule inhibition of a large, glycosylated, conformationally dynamic trimer [38,39]. From a therapeutic perspective, blocking the S–ACE2 interaction or the fusion process can prevent cell infection. Early in the pandemic, mAbs targeting the S protein receptor-binding domain (RBD) showed strong neutralising activity and clinical efficacy [40]. Several were approved for emergency use. For small-molecule discovery, S is a high-value but structurally and evolutionarily challenging target. The RBD and N-terminal domain (NTD) are among the most variable regions of the viral proteome, and variant turnover has repeatedly eroded the activity of single-epitope biologics, underscoring the ease of escape at exposed antigenic surfaces [41]. These constraints imply that durable S-directed small molecules, if achievable, will likely need to act through conserved, function-critical sites (particularly within S2), or allosteric mechanisms that stabilise non-productive conformational states of the prefusion trimer. Accordingly, in the context of small-molecule antivirals, S is best viewed as a selective opportunity, where the strongest near-term value may lie in structure-guided allosteric modulation and/or entry-pathway adjuncts that complement replication-directed therapy.

#### 2.1.2. Assays and Structural Biology

S alternates between “RBD-down” and “RBD-up” prefusion states to engage ACE2, then undergoes furin cleavage at S1/S2 and transmembrane serine protease 2 (TMPRSS2)- or cathepsin-dependent activation at S2′ prior to refolding into the post-fusion form. Notably, the S2 subunit is substantially more conserved than S1 but is metastable, functioning as a spring-loaded fusion machinery that transitions from the prefusion to post-fusion state upon receptor engagement and proteolytic activation, which both motivate S2-focused interventions and highlight the conformational challenge for durable inhibitor design [42]. Cryo-electron microscopy (cryo-EM) structures (e.g., PDB IDs 6VSB, 6VXX) define these conformational states and the RBD–ACE2 interface, while glycoproteomics has mapped a dense N-glycan shield across ~22 sites that shapes antigenicity and access to epitopes and small-molecule binding surfaces [37,43,44]. Several complementary assay systems are used to interrogate S-mediated entry. RBD–ACE2 binding can be quantified using surface plasmon resonance (SPR) or bio-layer interferometry (BLI). Lentiviral or vesicular stomatitis virus (VSV)-based pseudovirus neutralisation platforms are widely used to assess entry inhibition under lower-containment conditions, whereas authentic-virus assays, including plaque-reduction and focus-reduction neutralisation tests, provide more direct virological validation. Additional cell–cell fusion or syncytia readouts, such as split-luciferase assays, are useful for monitoring membrane-fusion inhibition. Finally, route-of-entry experiments comparing TMPRSS2-high and endosomal-entry systems help distinguish inhibitors of surface-entry pathways from those acting through cathepsin-dependent endosomal entry [45,46]. Importantly for small-molecule discovery, structural work has also revealed a free-fatty-acid pocket within the RBD that stabilises the closed trimer, highlighting an allosteric site that can be exploited to bias the conformational ensemble away from productive ACE2 engagement [47].

#### 2.1.3. Therapeutic Modalities

Most clinically advanced S inhibitors are biologics [48,49,50,51]. Nevertheless, several small-molecule strategies have been explored. First, small molecules that stabilise the prefusion trimer in a closed, ACE2-inaccessible state (“trimer-cavity binders”) provide a structure-guided route to allosteric entry inhibition [52]. These compounds have generally shown low-μM antiviral activity in cellular systems and should currently be regarded as proof-of-concept leads rather than as mature drug candidates. The key medicinal chemistry opportunity in this class is to convert conformational stabilisation into robust cellular potency while maintaining developability, given the large, dynamic, and glycosylated nature of the target surface. Second, various repurposed agents with entry-inhibiting potential have been identified. Among these, Arbidol (Umifenovir) is proposed to disrupt S-mediated fusion or stabilise prefusion states; however, it has shown only modest in vitro efficacy alongside inconsistent results in clinical trials [53]. While Arbidol (Figure 3) illustrates that small-molecule modulation of viral entry is possible, the overall quality and consistency of evidence do not currently support it as a strong benchmark for modern S-directed small-molecule campaigns. Third, entry can be inhibited indirectly through host-protease blockade of S priming. TMPRSS2 inhibitors (e.g., Camostat and Nafamostat) have supportive in vitro data but inconclusive clinical trial outcomes to date, while next-generation agents such as the peptidomimetic TMPRSS2 inhibitor N-0385 show nM potency and intranasal protection in animal models [54,55]. These approaches do not target S directly, but they remain relevant as small-molecule entry-pathway adjuncts that may be particularly useful in combination therapies.

*Note on non-small-molecule modalities:* Several potent entry blockers are peptides or protein-based ligands rather than small molecules, including heptad repeat 1 (HR1) and heptad repeat 2 (HR2) fusion-inhibitory peptides (e.g., EK1 and its cholesterol-conjugated derivative EK1C4) and de novo miniproteins (LCB1 series) [56,57]. These formats can achieve nM to pM neutralisation and intranasal protection in animal models, but they are discussed here only for mechanistic context and to clarify the current modality landscape.

Metal-based and inorganic compounds have also been explored as entry-directed agents, although this area remains much less developed than the organic small-molecule and biologic modalities discussed above. Screening of structurally diverse metallodrugs against the SARS-CoV-2 S–ACE2 interaction identified titanium-based complexes and polyoxometalates as inhibitors of viral attachment, with some polyoxometalates reaching high apparent potency in biochemical binding assays [58]. Gold-based compounds, including auranofin and related organometallic complexes, have also been reported to inhibit the S–ACE2 interaction in enzyme-linked immunosorbent assay (ELISA)-based assays, with half-maximal inhibitory concentration (IC_50_) values generally in the low- to mid-μM range [59]. However, these compounds often act through coordination chemistry, thiol reactivity, high charge density, or multivalent electrostatic interactions, and their selectivity, cellular target engagement, pharmacokinetics and antiviral translation remain less clearly established than for more conventional drug-like small molecules [58,59]. Therefore, metallodrugs currently provide useful mechanistic and chemical-biology leads for S-mediated entry inhibition and represent an interesting area for future investigation, although additional work is needed to establish their selectivity, cellular target engagement, PK, and translational potential for structural/accessory protein targeting.

#### 2.1.4. PK/PD and Clinical Data

De novo miniproteins (LCB1 series) achieve very high neutralisation activity and intranasal protection in mice but remain preclinical. Similarly, HR1/HR2 fusion-inhibitory peptides (EK1/EK1C4) have potent in vitro activity with intranasal efficacy in animals [60,61]. Additionally, structure-enabled trimer-cavity binders that stabilise the closed spike conformation show low-μM antiviral activity in cells (preclinical proof of concept). Furthermore, TMPRSS2 inhibition (e.g., N-0385) yields nM potency and intranasal protection in mice, but clinical validation is pending [62,63]. As a result, for small molecules, the key translational requirement is to demonstrate meaningful airway-relevant antiviral activity at exposures compatible with practical dosing and, ideally, early outpatient use. In particular, the pharmacology of entry inhibitors must align with the short window in which blocking new rounds of infection is maximally impactful.

#### 2.1.5. Resistance

Experience with anti-S mAbs illustrates the central resistance challenge for S-directed interventions: single-epitope pressure can be escaped rapidly, and deep mutational-scanning maps across the RBD and the NTD “supersite” identify numerous substitutions that ablate binding, several of which later emerged in variants of concern [64,65,66]. In immunocompromised hosts, prolonged infection under selective pressure from antibodies or convalescent plasma has repeatedly promoted within-host evolution, including S mutations that reduce neutralisation, underscoring the risks of monotherapy [67,68]. The same principles apply to small-molecule entry inhibitors. Durable strategies will likely need to target conserved, function-critical elements (particularly within S2) and/or be deployed as part of combination regimens to reduce the selective pressure concentrated on a single-entry epitope.

The major SARS-CoV-2 variants of concern illustrate how S evolution can influence the design of S-directed small molecules. Functionally and therapeutically relevant substitutions have clustered mainly in the NTD, RBD, furin-cleavage region, and selected S2-proximal sites. Alpha carried changes such as Δ69–70, N501Y and P681H; Beta and Gamma combined mutations including K417N/T, E484K and N501Y; Delta introduced L452R, T478K and P681R; and Omicron and its sublineages accumulated a much broader S mutational burden, including multiple RBD substitutions such as K417N, G446S, S477N, T478K, E484A, Q493R, Q498R, N501Y and Y505H, together with mutations around the S1/S2 and S2 regions [69,70,71]. These changes can alter ACE2 engagement, antigenicity, conformational equilibria, proteolytic activation, and fusion behaviour. For small-molecule design, this means that ligands directed at exposed or highly variable RBD/NTD surfaces are likely to require systematic testing across variant panels. By contrast, inhibitors targeting conserved S2 elements, internal allosteric pockets, or functionally constrained conformational transitions may offer greater potential for variant-resilient activity, although this must be demonstrated experimentally [69,72]. Host-directed entry inhibitors, such as TMPRSS2 inhibitors, are not directly affected by RBD or NTD substitutions, but their efficacy may still depend on variant-specific entry-route preferences [73].

#### 2.1.6. Development Status

As of 2026, small-molecule closed-state stabilisers remain at the lead-discovery/proof-of-concept stage. De novo mini-binders and S2 fusion-inhibitory peptides show potent neutralisation and intranasal protection in animal models, and remain preclinical [60]. Trimer-cavity stabilisers that lock S in closed prefusion states provide structure-guided leads with low-μM cellular activity, whereas host-protease inhibitors that block S priming represent an indirect small-molecule entry strategy with mixed clinical validation. In contrast, several biologic modalities (mAbs and related protein-based agents) have advanced furthest clinically, but their utility has been repeatedly constrained by variant susceptibility.

#### 2.1.7. Challenges and Opportunities

Variant turnover remains the central challenge for S as a durable antiviral target, particularly for interventions that engage RBD or NTD, which rapidly accumulate escape substitutions. Consequently, the most credible small-molecule opportunities are those that act through conserved, function-critical mechanisms (e.g., S2 fusion machinery) or exploit allosteric sites that stabilise non-productive conformations and may tolerate moderate sequence drift [66,74]. The S2 pathway is especially attractive for pan-coronavirus potential, but current high-potency activity in this region is dominated by peptides and biologics, highlighting an unmet need for truly small-molecule S2-directed chemical matter [75]. From a translational perspective, combination therapy is a practical hedge against rapid escape and compartmentalised pharmacodynamics. Pairing a rapid-onset entry blocker (direct or indirect) with a replication-directed antiviral can, in principle, reduce new cell infections while simultaneously suppressing replication in already infected cells, mirroring the established combination logic in antiviral therapy. Host-pathway adjuncts that block S protein activation, such as TMPRSS2 inhibitors, remain mechanistically compelling. However, randomised controlled trials of Camostat and Nafamostat have yielded inconclusive or negative results to date, while next-generation intranasal candidates such as N-0385 remain at the preclinical stage [54,76,77]. Finally, delivery remains an opportunity: intranasal or inhaled delivery strategies may maximise airway exposure where infection initiates, a feature that could be particularly valuable for entry-directed agents, provided that robust target engagement and antiviral effects can be demonstrated in physiologically relevant airway models.

### 2.2. Envelope (E) Protein

#### 2.2.1. Biology and Rationale

The E protein is a small (75 amino acids) viroporin that oligomerises as a homopentameric cation channel in membranes, a property defined at atomic resolution in lipid bilayers that mimic the endoplasmic reticulum–Golgi intermediate compartment (ERGIC) [79,80]. E contributes to virion assembly, budding, and release. It also modulates host stress and inflammatory pathways. In severe acute respiratory syndrome coronavirus (SARS-CoV), its ion channel activity transports Ca^2+^ and triggers NLRP3 inflammasome activation and interleukin (IL)-1β production [81,82]. Although not strictly essential for coronavirus replication in vitro, E is a key virulence factor: deletion of E attenuates SARS-CoV in animal models, and mutations that disrupt channel activity reduce pathogenicity [83,84]. Mechanistically, E localises to the ERGIC, where budding occurs and can influence S/M protein maturation and particle assembly. Its C-terminal postsynaptic density protein 95/discs large/zonula occludens-1 (PDZ)-binding motif also engages host protein associated with Lin Seven 1 (PALS1), a junctional scaffold implicated in epithelial barrier integrity [14,85].

#### 2.2.2. Assays and Structural Biology

**Figure 4:**
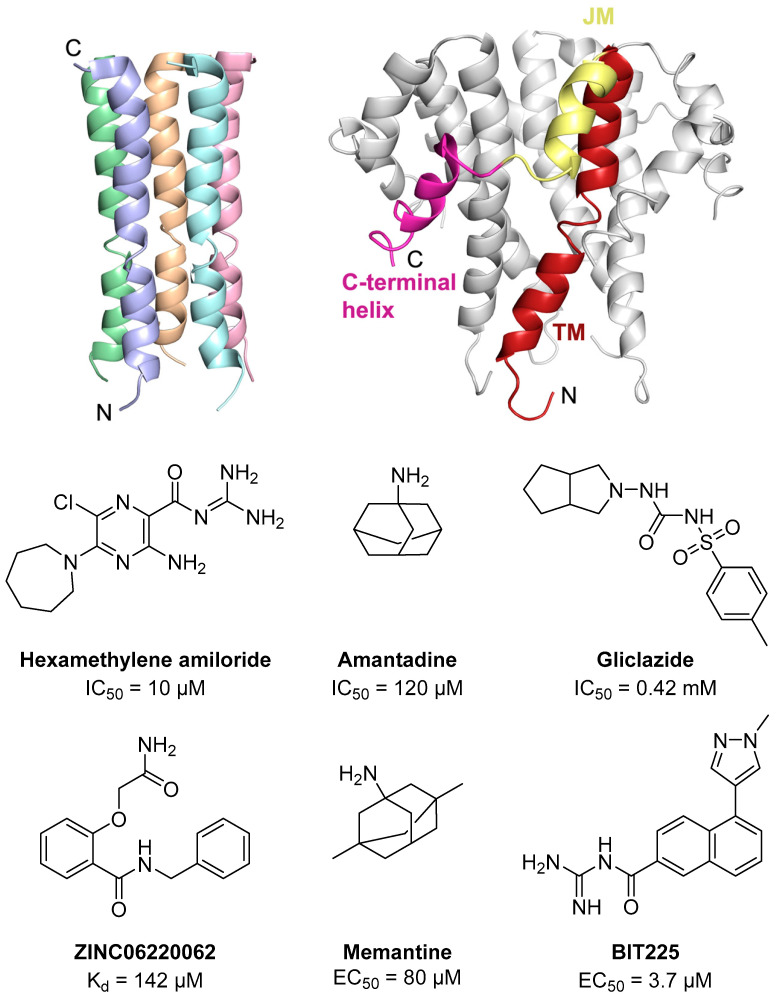
(**Top left**): NMR structure of the SARS-CoV-2 E transmembrane E8-R38 (TM) domain (PDB ID 7K3G). The five protomers of the helical bundle are shown in purple, cyan, pink, beige, and green. C and N indicate the C- and N-termini, respectively. (**Top right**): NMR structure of the SARS-CoV E protein (PDB ID 5X29). The TM domain E8-R38 is shown in red, the juxtamembrane middle helical segment (JM) L39-S50 in yellow, and the C-terminal helix L51-L65 in magenta, while the other four protomers are shown in grey. (**Bottom**): Reported and putative E-channel inhibitors, including hexamethylene amiloride (HMA) [92], amantadine [94], gliclazide [95], ZINC06220062 [89], memantine [96], and BIT225 (an investigational viroporin inhibitor) [91].

Solid-state nuclear magnetic resonance (NMR) of the SARS-CoV-2 E transmembrane domain in ERGIC-like bilayers shows a five-helix bundle that forms a narrow, druggable pore (Figure 4). This structural arrangement enables direct mapping of small-molecule binding sites and channel-blocking mechanisms [79]. Functional assays reconstituting E in lipid bilayers quantify ion conductance and selectivity, including Ca^2+^ flux linked to NLRP3 activation, while cell-based readouts (e.g., virus-like particle release and co-expression with M/S) report on assembly/egress functions [14,82]. Subcellular localisation is supported by defined targeting signals in E’s cytoplasmic tail that direct it to the Golgi/ERGIC, consistent with its role at the budding site [85].

#### 2.2.3. Chemical Matter

The E protein has long been considered a potential viroporin target, with early work showing that hexamethylene amiloride (HMA) (Figure 4) blocks E-channel conductance of human coronavirus (HCoV)-229E and Mouse Hepatitis Virus (MHV) in bilayers and reduces viral replication in cells, establishing tractability for small-molecule channel blockers [86]. More recently, Amantadine and HMA were shown to inhibit SARS-CoV-2 E ion-channel activity in cell-based reconstitution and electrophysiology assays, while Rimantadine was inactive against SARS-CoV-2 E, confirming that E is druggable with repurposed chemotypes, albeit with modest potencies [87]. Orthogonal, bacteria-based assays likewise identified Gliclazide and Memantine as E-channel blockers and linked channel inhibition to reduced SARS-CoV-2 replication, further validating E as a pharmacological target [88]. Structure-guided efforts have begun to move beyond repurposing: an NMR ligand-screen campaign targeting the C-terminal helical bundle of E detected small-molecule binders (e.g., “ligand 3: ZINC06220062”) with an equilibrium dissociation constant (K_d_) of 142 μM, providing tangible fragment-like starting points for optimisation against an experimentally supported epitope [89,90].

A notable advance is BIT225, a clinical-stage viroporin inhibitor developed for human immunodeficiency virus type 1 (HIV-1) viral protein U (Vpu) and Hepatitis C Virus p7, that inhibits SARS-CoV-2 E channel activity and shows broad-spectrum antiviral activity in Vero and Calu-3 cells against multiple strains. Selectivity was demonstrated in oocytes (no inhibition of Anoctamin-1), supporting an on-target mechanism [91]. Solid-state NMR has begun to clarify drug–channel binding modes in SARS-CoV-2 E (e.g., HMA binds one per pentamer at the protein–lipid interface), offering structure-level hypotheses to guide medicinal chemistry [92].

#### 2.2.4. PK/PD and Safety

Amantadine and related cationic amines have favourable oral PK and central nervous system (CNS) penetration from historical use, but their anti-E potencies are in the low- to mid-μM range, implying that clinically achievable exposures may be insufficient for reliable antiviral efficacy as monotherapy [87]. HMA demonstrates antiviral proof-of-concept but is not a clinical candidate, and recent biophysical work suggests membrane-interfacial binding, raising off-target risk at higher exposures [86,92]. By contrast, BIT225 has an established human safety and PK record from HIV and Hepatitis C Virus trials (Phase I/II), offering a potential fast-follower path for E-targeted therapy if clinical efficacy against coronavirus disease 2019 (COVID-19) is demonstrated [93]. From a class-safety perspective, E-channel inhibitors must be screened broadly against human ion channels and membrane-active liabilities. Nonetheless, mechanistic selectivity (e.g., lack of Anoctamin-1 block for BIT225) and structure-enabled design around the E lumen and interface provide credible routes to therapeutic windows [91].

#### 2.2.5. Development Status

As of 2026, E-targeted antiviral inhibitors are in the hit-to-lead stage. Repurposed blockers (e.g., Amantadine and HMA) serve as tool compounds but lack clinical validation for COVID-19 [87]. BIT225 remains the most advanced E-inhibitor with anti-SARS-CoV-2 activity, supported by robust cellular efficacy and selectivity. It is a clinical-stage drug from other viroporin programmes, and preclinical results support progression to COVID-19 studies [91]. However, recently, in a randomised, double-blind Phase 2 trial in Thailand (Trial ACTRN12623000035628), BIT225 met safety and tolerability but did not meet the primary virologic endpoint (change in nasal SARS-CoV-2 load in days 1–7 versus placebo); time to sustained recovery and clinical improvement were also similar. When dosed 200/400 mg once daily (QD) for seven days, the study saw no primary or key secondary efficacy benefit; an exploratory days 3–9 signal (*p* = 0.02) was noted but was hypothesis-generating only.

#### 2.2.6. Challenges and Opportunities

Potency and selectivity are the key hurdles: many first-wave hits are μM and membrane-active, necessitating structure-guided optimisation (helped by NMR structures of E pentamers and drug-binding site mapping) [88,92]. Demonstrating clinically meaningful viroporin PD in the ERGIC/Golgi compartment is non-trivial, but cellular and organotypic assays now exist to bridge ion-flux readouts to viral egress and pathogenesis [88]. From a resistance standpoint, the E channel is functionally constrained, and many E mutations are attenuating, suggesting a higher barrier to resistance than for entry-neutralising epitopes. Earlier coronavirus studies showed E deletions or channel-dead mutations markedly impair virulence and assembly [86]. Finally, polypharmacology opportunities are attractive: agents that occlude the pore or stabilise non-conductive oligomers could be combined with inhibitors of E–PDZ interactions (e.g., PALS1 axis) to couple antiviral effects with mitigation of E-driven inflammation, an avenue supported by the demonstrated PDZ-binding motif and host-pathway data [89].

### 2.3. Membrane (M) Protein

#### 2.3.1. Biology and Rationale

The M protein is the most abundant structural protein (222 amino acids) and is a central organiser of virion morphogenesis. It scaffolds the envelope, drives budding at the ERGIC, and coordinates assembly via multiprotein contacts with S, N, and E. Single-particle cryo-EM of SARS-CoV-2 M resolved a 50 kDa homodimer in lipid nanodiscs at 3.5 Å. The fold is structurally related to the accessory protein ORF3a, consistent with a matrix-like role beneath the envelope [97]. Complementary work captured two M conformers (“long” and “short”), and cryogenic electron tomography and biochemistry map M–N–RNA contacts, supporting a model in which M dimers oligomerise, shape ERGIC membranes and recruit the ribonucleoprotein (RNP) during budding [98,99]. Beyond morphogenesis, M antagonises type I IFN signalling by disrupting assembly of the retinoic acid-inducible gene I (RIG-I)/melanoma differentiation-associated protein 5 (MDA5)–mitochondrial antiviral-signalling protein (MAVS)–tumour necrosis factor receptor-associated factor 3 (TRAF3)/TANK-binding kinase 1 (TBK1) axis and impairing interferon regulatory factor 3 (IRF3) activation, indicating a conserved role in immune evasion [100,101].

#### 2.3.2. Assays and Structural Biology

**Figure 5:**
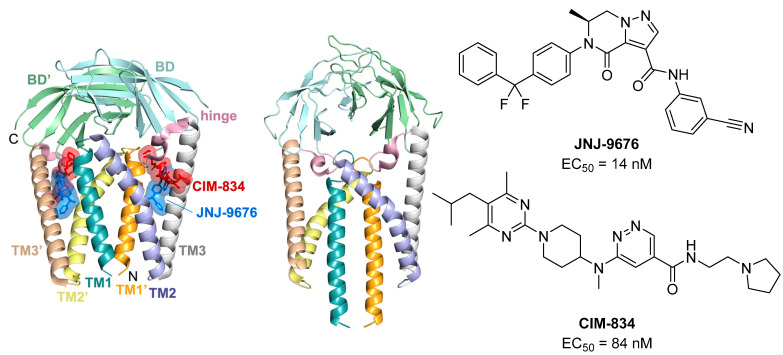
(**Left**): Cryo-EM structure of the SARS-CoV-2 M protein dimer in the short form, with bound CIM-834 (red; PDB ID 9EXA), and JNJ-9676 (blue; PDB ID 8W2E) superposed for comparison. TM1 (L17-N41), TM2 (R42-I73), and TM3 (N74-R105) in the first and second monomers are shown in teal, purple, grey, orange, yellow, and beige. The hinge regions (T106-T116) are shown in pink. The C-terminal β-sheet domains (BDs) (N117-Y204) of the two monomers are shown in cyan and green. (**Centre**): Cryo-EM structure of the SARS-CoV-2 M protein dimer in the long form (PDB ID 7VGR), with the regions coloured as in the short form. Bound antibodies are not included in the figures. (**Right**): Representative small-molecule scaffolds explored for M modulation: JNJ-9676 [106] and CIM-834 [107]. Both compounds exhibit double-digit nM potency against SARS-CoV-2.

Single-particle cryo-EM resolved SARS-CoV-2 M in nanodiscs, showing a homodimer (“mushroom” shape) built from two domain-swapped three-helix bundles in the membrane and two cytosolic domains (Figure 5). Conserved hinge elements likely mediate assembly-linked conformational changes [97,98]. This architecture rationalises a membrane-scaffolding function distinct from ion channels [97]. For function, interaction assays, such as co-immunoprecipitation (co-IP), split reporters, and bimolecular fluorescence complementation, quantify M–N and M–S contacts. Recent experiments demonstrate that N’s C-terminal region and linker drive packaging with M and RNA [102]. Virus-like particle (VLP) systems offer a phenotypic assembly readout: in coronaviruses, M and E can be sufficient for VLPs, with N enhancing RNP incorporation and S contributing to Spike maturation and retention, an approach now widely used to screen for assembly disruptors [103,104,105].

#### 2.3.3. Chemical Matter

A first-in-class, direct-acting M inhibitor, JNJ-9676 (Figure 5), exhibits double-digit nM potency against SARS-CoV-2 (including Omicron BA.1 and Delta), SARS-CoV, and several zoonotic sarbecoviruses (e.g., WIV-1, SHC014, and Pangolin coronavirus), with weaker activity against other β-coronaviruses and no activity against common α-coronaviruses. In primary human nasal epithelium (air–liquid interface), JNJ-9676 reduced viral RNA concentrations with a half-maximal effective concentration (EC_50_) of 94 nM and a 90% effective concentration (EC_90_) of 132 nM, comparable to Nirmatrelvir in the same system. Time-of-addition and replication assays indicated a late assembly and egress mechanism. Cryo-EM of M in complex with JNJ-9676 revealed a ligand-induced pocket at the dimer transmembrane interface, and compound binding stabilises an altered state between the canonical M-long and M-short conformers, rationalising the block in infectious particle formation. In vitro resistance selection maps escape-associated substitutions to the M dimer interface, thereby providing genetic validation of the target [106].

An independent chemotype, CIM-834, emerged from a 350,000-compound phenotypic screen followed by optimisation. CIM-834 inhibits SARS-CoV-2 variants with EC_50_ values of 84–112 nM (A549-ACE2/TMPRSS2 and green fluorescent protein-expressing Vero E6 cells), retains activity against SARS-CoV, and shows reduced or absent activity against Middle East respiratory syndrome coronavirus (MERS-CoV) and α-coronaviruses. Primary human nasal air-liquid interface (ALI) cultures showed 4–5 log_10_ reductions in virus yield at low-μM concentrations. Mechanistically, CIM-834 acts late: it does not affect RNA synthesis in single-cycle assays but blocks the formation of infectious particles, disrupts M oligomerisation, and inhibits VLP release. Cryo-EM places the inhibitor between transmembrane segment 2 (TM2) and TM3 and the conserved hinge (residues 106–116 of M), stabilising M-short and preventing the short-to-long conformational switch required for budding. Resistance mapping identifies mutant P132S of M as a determinant of escape. For instance, substitutions such as M91K and N117K confer only modest EC_50_ shifts, consistent with the hinge and interface mechanism [107].

#### 2.3.4. PK/PD and Safety

In Syrian hamster models, oral JNJ-9676 administered pre-exposure at 25 mg kg^−1^ twice daily (BID) reduced lung viral RNA by approximately 3.5 log_10_ and infectious virus by approximately 4 log_10_, with concomitant normalisation of lung histopathology. In post-exposure studies, efficacy remained significant when dosing at 75 mg kg^−1^ BID was initiated 48 h after infection. Together with the ALI data, these results demonstrate on-target pharmacodynamics for an M-directed assembly inhibitor and support oral developability (human PK/PD pending) [106].

For CIM-834, oral dosing showed good bioavailability, favourable lung distribution, and robust efficacy in severe combined immunodeficiency (SCID) mice and Syrian hamsters, including reduction in lung infectious titres by approximately 3–4 log_10_ and, importantly, prevention of transmission to sentinels in hamsters under study conditions. Ritonavir boosting was used in hamsters for PK support. These in vivo data independently validate M-target engagement and late-stage PD [107].

#### 2.3.5. Development Status

Two preclinical M-targeting series with in vivo proof of concept have now been disclosed: JNJ-9676 and CIM-834. Both show direct binding to purified M, resistance mutations mapping to the dimer and hinge interface, potent cellular antiviral activity including in primary airway epithelia, and oral efficacy in animal infection models [106,107]. To date, no M-targeted agent has entered human clinical trials. Within the structural and accessory proteome reviewed here, M currently represents the most advanced and translationally compelling small-molecule target.

#### 2.3.6. Challenges and Opportunities

The conformational plasticity of the M dimer (short and long states) suggested an “undruggable” surface. However, structure-enabled campaigns have revealed ligand-induced pockets and conformation-locking as practical strategies. Notably, CIM-834 (P132S escape) and JNJ-9676 (overlapping interface substitutions) map to distinct but convergent regions of the dimer and hinge interface, indicating non-overlapping resistance pathways and motivating within-target combinations to increase the genetic barrier. The high conservation of M across sarbecoviruses (i.e., SARS-CoV and SARS-CoV-2) supports pan-sarbecovirus potential and makes it an attractive combination partner for the main protease (M^pro^; Nsp5) and the RNA-dependent RNA polymerase (RdRp; Nsp12) inhibitors, providing an orthogonal and largely variant-agnostic mechanism of action. Prospective work should quantify fitness costs of M-interface substitutions, extend ERGIC-aware phenotypic assays, and track surveillance for rare interface variants as programmes advance [106,107].

### 2.4. Nucleocapsid (N) Protein

#### 2.4.1. Biology and Rationale

The SARS-CoV-2 N protein is a 419-residue multidomain RNA-binding protein that packages the ~30 kb genome into ribonucleoprotein (RNP) assemblies and supports replication and virion assembly. It comprises an N-terminal RNA-binding domain (NTD) and a C-terminal dimerisation/RNA-binding domain (CTD) linked by a Ser/Arg-rich intrinsically disordered region/linker region (IDR/LKR). Both NTD and CTD present basic grooves that engage single-stranded/double-stranded RNA (ss/dsRNA), with high-resolution structures (e.g., PDB ID 6YUN, 7CE0, and 6ZCO) defining conserved, positively charged pockets for RNA interaction [98,99,108]. N undergoes RNA-driven liquid–liquid phase separation (LLPS), forming condensates that are thought to concentrate viral components and scaffold replication and packaging. LLPS is chemically tuneable in vitro (e.g., by 1,6-hexanediol, lipoic acid, and Kanamycin), underscoring tractability [109,110]. The protein also interfaces with host pathways, including stress-granule biology and innate immune signalling, and its functions are tightly regulated by phosphorylation within the Ser/Arg-rich linker, which modulates RNA affinity, oligomerisation and condensate behaviour, while creating phospho-motifs recognised by 14-3-3 proteins [111,112]. During assembly, the CTD of N engages the cytosolic domain of M protein to recruit N–RNA to budding membranes, rationalising protein–protein-interface (PPI) disruption as a complementary antiviral strategy; VLP systems recapitulate the M–N dependency in coronavirus assembly [98,99].

#### 2.4.2. Assays and Structural Biology

**Figure 6:**
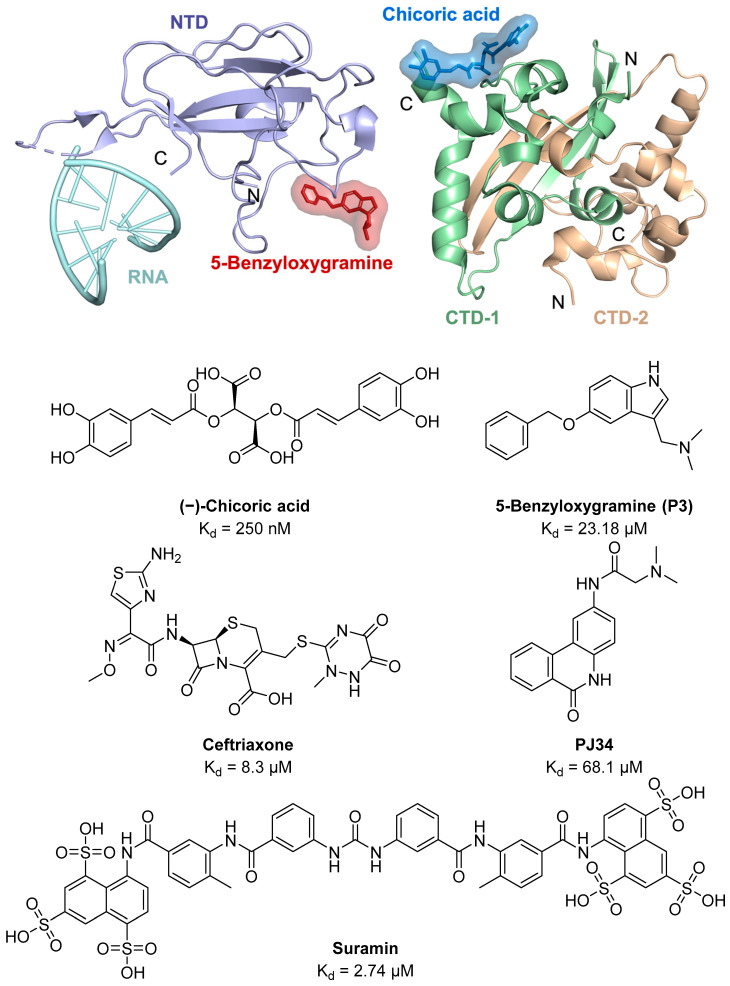
(**Top left**): Crystal structure of the SARS-CoV-2 N protein N-terminal domain (NTD) in complex with 5-benzyloxygramine (PDB ID 8IV3), with RNA (cyan; PDB ID 7XWZ) superposed for comparison. The NTD and 5-benzyloxygramine are shown in purple and red, respectively. (**Top right**): Crystal structure of the SARS-CoV-2 N protein C-terminal domain (CTD) dimer in complex with chicoric acid (PDB ID 7UXZ). The two N-CTD monomers are shown in green and beige, and chicoric acid is shown in blue. (**Bottom**): Representative small-molecule modulators reported to affect N, including (−)-Chicoric acid [117], 5-Benzyloxygramine [126], Suramin [127], Ceftriaxone [121], and PJ34 [128].

Structural work has defined an NTD β-sheet core with a basic β-hairpin forming the primary RNA-binding pocket and a strand-swapped CTD dimer with basic surfaces that also bind RNA (Figure 6). These features guide ligand design against the NTD cleft and CTD dimer interface [113]. Biochemical readouts include fluorescence-polarisation or electrophoretic mobility shift assay (EMSA) for RNA binding (isolated NTD/CTD and full-length N) [114], and biophysical/biochemical assays for PPIs (e.g., 14-3-3 engagement of defined phospho-motifs; M–N recruitment in co-expression/VLP systems). Because full-length N is highly dynamic and forms RNA-driven condensates, LLPS assays (turbidity and fluorescence microscopy) provide orthogonal, function-proximal readouts that complement RNA-binding and dimerization assays. Small molecules that perturb LLPS offer an additional screening and mode of action validation axis [115,116].

#### 2.4.3. Chemical Matter

Multiple orthogonal campaigns have demonstrated that the SARS-CoV-2 N protein is ligandable at both its RNA-binding surfaces and allosteric sites. A high-throughput fluorescence-polarisation screen (more than 3200 bioactives) identified Chicoric acid (Figure 6) as a CTD-binding modulator that disrupts the association of N protein with RNA. Crystallography revealed a 1.7 Å CTD–Chicoric acid complex, defining a shallow pocket adjacent to the basic RNA groove with a K_d_ of 250 nM by isothermal titration calorimetry (ITC), and showing reduced viral replication in cell culture, thus delivering the first small-molecule structure of N and a validated allosteric site for structure-guided optimisation [117]. Polyanionic scaffolds also inhibit N–RNA engagement. Suramin binds the NTD, perturbs N–RNA interactions, and interferes with packaging and condensate functions in vitro and in cells, although its size and promiscuity limit drug potential. These studies nonetheless pharmacologically validate N-targeting at the NTD surface [118,119].

Repurposing-led and structure-guided efforts provide complementary support. Structural biology studies report Ceftriaxone binding to NTD, with biophysical evidence that it blocks RNA binding and inhibits RNP assembly in vitro; useful as a mechanistic probe [120,121]. Additional small-molecule hits that disrupt the N–RNA interface have emerged from biochemical assays, including “G12”, an N–RNA interaction blocker with effects on N assemblies and LLPS, adding diverse starting points for medicinal chemistry [122]. Pre-pandemic coronavirus work remains instructive: PJ34 (HCoV-OC43) and 5-benzyloxygramine (“P3”) (MERS-CoV) target NTD interfaces to inhibit RNA binding or induce non-native oligomerisation. Despite virological distance, these chemotypes map conserved basic pockets and PPI surfaces that informed SARS-CoV-2 targeting [123,124].

#### 2.4.4. PK/PD and Safety

Most first-wave N ligands are highly polar (polyphenols, polysulphonates) and show sub-μM biochemical but modest cellular potency, foreshadowing permeability and clearance challenges (e.g., Chicoric acid and Suramin) [117,118]. None have been reported to have in vivo pharmacokinetics against SARS-CoV-2. Suramin’s known systemic liabilities and Chicoric acid’s polyphenolic character argue for prodrug or local (e.g., inhaled) delivery concepts if such chemotypes are pursued. From a selectivity standpoint, N lacks a close human orthologue. However, polyanions and nucleic-acid-interacting dyes may exhibit off-target binding to host RNA-binding proteins (RBPs) and RNA, limiting their selectivity and developability; therefore, broad panel screening and condensate-aware cell assays are essential before animal studies [125].

#### 2.4.5. Development Status

As of 2026, N targeting antiviral programmes largely remain at the hit-to-lead stage. The most advanced SARS-CoV-2 data comprise validated tool compounds (e.g., Chicoric acid co-crystal; Suramin as NTD binders) and biophysical/structural probes (e.g., NTD–Ceftriaxone complex), alongside increasing mechanistic understanding of N LLPS and assembly that enables target-engagement readouts in cells [117,118]. Authoritative reviews emphasise the opportunity but also the current preclinical stage of N chemical biology [125].

#### 2.4.6. Challenges and Opportunities

Associated challenges include targeting multivalent, highly basic RNA-binding surfaces and intrinsically disordered regions that drive LLPS, which often necessitate either highly charged ligands with poor PK properties or finely tuned allosteric or PPI binders. Moreover, phase-separation phenotypes can confound nonspecific modifiers, necessitating orthogonal assays (e.g., biochemical RNA displacement, LLPS microscopy and/or VLP/M–N recruitment) [124].

Opportunities include the exploitation of allosteric CTD pockets (e.g., the Chicoric acid site of action) that provide structure-enabled vectors for potency and drug-likeness without competing with RNA phosphates directly. This site is partly conserved across β-coronaviruses, supporting breadth [117]. In addition, the NTD RNA groove and adjacent basic patches are ligandable (Suramin/Ceftriaxone), supporting fragment-to-lead campaigns for compact anionic or H-bond-rich chemotypes [118]. Furthermore, targeting assembly interfaces (e.g., M–N recruitment) offers a complementary mechanism to polymerase or protease inhibitors, potentially collapsing infectious yield even with partial inhibition. Finally, N is function-constrained and relatively conserved compared with Spike, which may slow resistance pathways. Phosphorylation-dependent regulation and 14-3-3 protein engagement add exploitable host-PPI nodes for adjunct strategies [125].

## 3. Accessory Proteins as Potential Targets

SARS-CoV-2 encodes eleven accessory proteins: ORF3a, ORF3b, ORF3c, ORF3d, ORF6, ORF7a, ORF7b, ORF8, ORF9b, ORF9c, and ORF10 [129]. Accessory proteins are dispensable for basic replication in standard cell culture, yet play significant roles in immune evasion, virulence, and tissue tropism through targeted rewiring of host pathways [130,131]. Collectively, they act as IFN antagonists and modulators of membrane trafficking and inflammatory signalling, although their contribution to viral fitness and pathogenesis is often context-dependent [20,132]. From a therapeutic standpoint, accessory proteins are attractive adjunct targets: inhibiting them is unlikely to sterilise infection on its own but could dearm immune evasion and reduce pathology, especially in combination with DAAs. Their host-interface mechanisms (e.g., ORF6–Nup98/Rae1, ORF7a–BST-2, and ORF9b–TOM70) create PPI surfaces that may be tractable to small molecules or biologics. The chief challenges are sequence variability, context-dependent phenotypes and ensuring on-target readouts in physiologically relevant systems [20].

### 3.1. ORF3a: Viroporin and Virulence Factor

#### 3.1.1. Biology and Rationale

Within the ORF3 region, ORF3a should be distinguished from the smaller overlapping alternative-frame ORFs ORF3b, ORF3c, and ORF3d. Unlike ORF3a, these overlapping ORFs are not supported by comparable structural, trafficking, or pharmacological evidence. They are discussed mainly in the context of immune modulation and remain less mature from the standpoint of direct small-molecule tractability [131,133].

**Figure 7:**
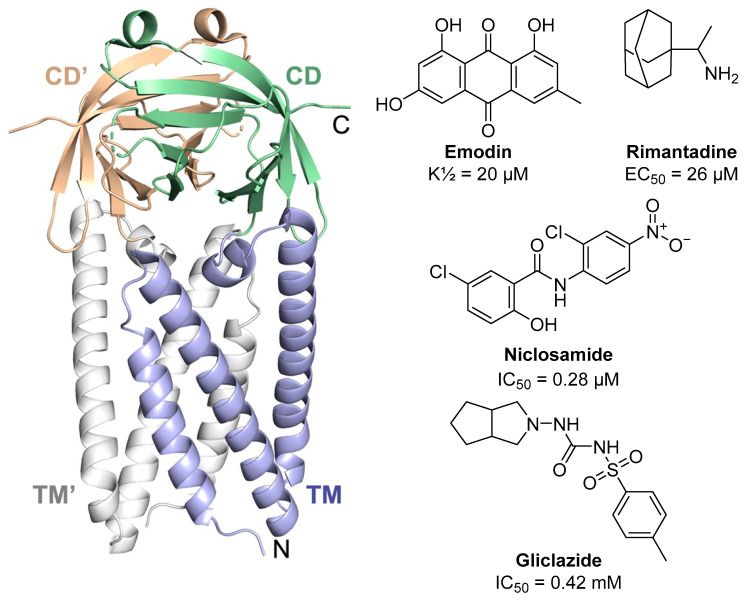
(**Left**): Cryo-EM structure of the SARS-CoV-2 ORF3a dimer (PDB ID 7KJR). The transmembrane regions (TMs) S40-A143 of the two subunits are shown in purple and grey, and the cytosolic domains (CDs) N144-D238 are coloured in green and beige. Bound membrane scaffold protein 1E3D1 and 1,2-dioleoyl-sn-glycero-3-phosphoethanolamine (DOPE) lipids are not included in the figure. (**Right**): Representative small molecules reported to modulate ORF3a/viroporin activity: Emodin [142], Rimantadine [96], Niclosamide [149], and Gliclazide [95].

ORF3a is a 275-amino-acid multipass membrane protein that localises to endo-lysosomal and Golgi membranes and is strongly implicated in virion egress and immune modulation. A 2.1 Å cryo-EM structure revealed a dimer/tetramer architecture with a polar cavity and putative ion-conduction pathways (Figure 7). Reconstitution in liposomes showed Ca^2+^-permeable, non-selective cation flux and sensitivity to polycationic blockers, supporting a viroporin-like function [134]. ORF3a has been reported to prime and activate the NLRP3 inflammasome by promoting nuclear factor kappa B (NF-κB)-dependent pro-interleukin-1 beta (pro-IL-1β) induction, potassium ion (K^+^) efflux, and apoptosis-associated speck-like protein containing a caspase recruitment domain (ASC)/caspase-1 activation. In human cells, ORF3a can also trigger interleukin-1 beta (IL-1β) maturation, linking it to COVID-19-associated hyperinflammation [23,135]. It has also been reported to block autophagosome–lysosome fusion by engaging the homotypic fusion and vacuole protein sorting (HOPS) tethering machinery, perturb Ras-related protein Rab-7a (Rab7) and lysosomal dynamics, and promote lysosomal exocytosis, thereby enhancing virion release [136,137]. Genetic studies in vivo indicate that accessory proteins, including ORF3a, contribute substantially to pathogenesis in keratin 18-human ACE2 (K18-hACE2) mice. Moreover, multi-accessory-gene deletion viruses, including ΔORF3a, are markedly attenuated yet immunogenic, underscoring the role of ORF3a as a virulence factor [138,139].

It is important to note that the ion channel interpretation is debated. Some studies report that ORF3a does not form a classical cation channel (and instead modulates lysosomal water and acidification), so therapeutics may need to address both pore-blocking and trafficking- and lysosome-centric mechanisms [140,141].

#### 3.1.2. Assays and Structural Biology

Structural work using cryo-EM defines the druggable cavities and oligomer interfaces in ORF3a. Functional assessment can then be performed across complementary assays. Channel activity is typically measured using liposome ion flux assays or patch-clamp recordings in heterologous systems. In parallel, inflammasome-related effects can be evaluated through established readouts of activation, including changes in pro-IL-1β expression, caspase-1/ASC speck formation, and IL-1β processing. Likewise, effects on autophagy and lysosomal trafficking can be monitored using markers of autophagic flux, membrane-fusion machinery, Rab7-dependent trafficking, lysosomal membrane exposure, and lysosomal pH [23,134]. These orthogonal assays are important given the ongoing debate over the precise transport function of ORF3a and help ensure that putative hits are validated across channel-, trafficking-, and inflammasome-centred phenotypes [141].

#### 3.1.3. Chemical Matter

Emodin, an anthraquinone (Figure 7), inhibits the SARS-CoV ORF3a channel with a half-maximal blocking concentration (K½) of 20 µM, and reduces HCoV-OC43 release [142]. For SARS-CoV-2 ORF3a specifically, recombinant-system electrophysiology shows inhibition by classical viroporin blockers (e.g., Amantadine and Rimantadine) and with several flavonoids and phenolics, with correlation between channel block and reduced ORF3a-induced cytotoxicity [143]. The structure also revealed sensitivity to polycationic species, which is consistent with pore blocking, nominating cationic scaffolds as tractable starting points [134].

Niclosamide, a protonophore with endo-lysosomal effects, inhibits SARS-CoV-2 in vitro and has been clinically explored [144,145]. While not ORF3a-selective, its ability to neutralise acidic compartments and perturb exocytosis aligns mechanistically with ORF3a-driven lysosomal egress and autophagy blockade. In a proof-of-concept “nasal swab” assay, Emodin and Gliclazide reportedly suppressed ORF3a and E channel signals across variants. Although methodologically unconventional, it reinforces on-target tractability for small molecules at mucosal surfaces [95]. Finally, at least one study failed to detect bona fide cation conductance attributable to ORF3a, highlighting potential system-dependent artifacts. Chemical campaigns should therefore include lysosome-function and inflammasome assays, not only ion flux [141].

#### 3.1.4. PK/PD and Safety

Amantadine and Rimantadine have well-characterised oral PK but limited antiviral exposure margins for COVID-19. Their lack of selectivity raises on-target and off-target concerns [143]. Niclosamide is poorly bioavailable orally and inhaled or nebulised formulations are being tested to achieve airway concentrations compatible with its endo-lysosomal pharmacology [146]. For any future ORF3a-directed agent, distribution to the endo-lysosomal and Golgi compartments of airway epithelium and minimisation of host-channel liabilities (e.g., cation transporters and/or lysosomal homeostasis) will be the key PD and safety gates [147].

#### 3.1.5. Development Status

ORF3a-directed small-molecule discovery remains at an early, preclinical proof-of-concept stage, and current chemical matter is largely limited to repurposed or non-selective modulators whose on-target relevance still requires careful validation. Multiple repurposing trials, including studies of niclosamide and inhaled formulations, have reported mixed outcomes, and clinical efficacy remains unproven [146]. In contrast, ORF3a is being leveraged for live-attenuated vaccine design: deletion of accessory genes, including ORF3a, yields robust attenuation with preserved immunogenicity in animal models [138].

#### 3.1.6. Resistance

ORF3a is polymorphic (e.g., Q57H among common variants); however, several functions, such as inflammasome activation and lysosome and exocytosis control, are retained across isolates. As ORF3a is not strictly essential for replication in cell culture, escape via mutation or loss is possible. However, in vivo, such changes tend to attenuate disease, implying a favourable resistance trade-off for pathogenesis-focused therapeutics [139,148].

#### 3.1.7. Challenges and Opportunities

The central challenge is mechanistic heterogeneity, as data support both ion transport activity and lysosome/trafficking control, and some groups do not observe canonical cation currents. This argues for poly-assay development funnels that include lysosomal pH, acidification, exocytosis, autophagy flux, and inflammasome outputs alongside ion-flux [136,141]. Several therapeutic opportunities remain open in the ORF3a space. These include structure-guided pore-blocking chemotypes informed by high-resolution ORF3a structures and legacy Emodin and Adamantane SAR, as well as lysosome-directed modulators that reverse ORF3a-driven deacidification and exocytosis. In parallel, anti-inflammatory adjuncts that dampen NLRP3 inflammasome activation may offer a complementary strategy for reducing downstream pathology. Combination strategies with E-protein inhibitors (two viroporins) or with replication inhibitors may yield additive reductions in infectious virus output [134,143].

### 3.2. ORF6: Interferon Antagonist

#### 3.2.1. Biology and Rationale

ORF6 is a small accessory protein (61 amino acids) that localises to the ER/Golgi and nuclear pore interface, where it potently antagonises IFN signalling by binding the Nup98–Rae1 complex and blocking Karyopherin-mediated nuclear import of STAT1, STAT2, and other transcription factors. Mechanistically, its C-terminal domain engages a basic groove on Rae1. Mutational analyses (e.g., M58R) abrogate Nup98–Rae1 binding and IFN antagonism. Viruses lacking ORF6 (often in combination with other accessory ORFs) are markedly attenuated in immune-competent models, underscoring its contribution to pathogenesis [138,150,151]. While ORF6 is widely recognised as a dominant IFN antagonist, some studies in differentiated respiratory cells report context-dependent effects and limited sufficiency of ORF6 alone, highlighting the importance of cell type and expression level [152].

#### 3.2.2. Assays and Structural Biology

**Figure 8:**
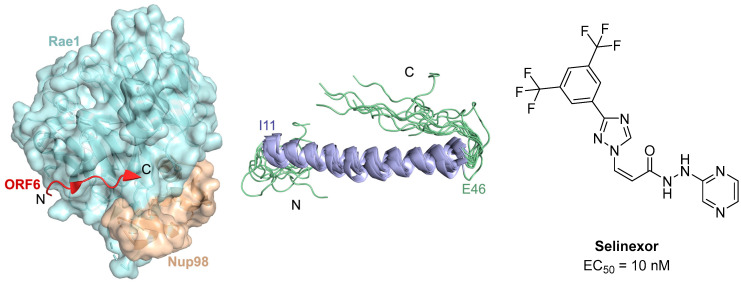
(**Left**): Crystal structure of the Rae1–Nup98 complex, with Rae1 shown in cyan, Nup98 in beige, and the bound C-terminal peptide (D53-D61) of SARS-CoV-2 ORF6 shown in red (PDB ID 7F60). (**Centre**): Ensemble of ten SARS-CoV-2 ORF6 models derived from NMR chemical shifts using CS-Rosetta (PDB ID 9A8Y). The α-helical segment (I11–T45) is shown in purple, whereas the N-terminal region (M1–T10) and the flexible C-terminus (E46–D61) are shown in green. (**Right**): Selinexor [159], an XPO1 inhibitor explored as an indirect countermeasure to ORF6 activity.

Although obtaining full-length structures has remained challenging, two advances now provide a foundation for medicinal chemistry: First, crystal structures of Rae1–Nup98 in complex with C-terminal peptides of ORF6 (2.4–2.9 Å) define the drug-targetable PPI interface (Figure 8) [27]. Second, NMR in proteoliposomes resolves an α-helical ORF6 topology with a rigid N-terminal segment and flexible C-terminus that mediates host binding [133].

Functional readouts include nuclear import reporters assessing STAT1 and interferon-stimulated gene factor 3 (ISGF3) translocation, bulk mRNA export assays, and co-IP and biophysics for ORF6–Nup98/Rae1 engagement. Quantitative imaging shows SARS-CoV-2 ORF6 is substantially more potent than SARS-CoV ORF6 in inhibiting nuclear import and mRNA export [28,153,154].

#### 3.2.3. Chemical Matter

Although direct ORF6 inhibitors have not yet been reported, tractable strategies, such as interface-guided inhibitors and peptides, are emerging. For instance, the Rae1–Nup98:ORF6 co-crystal structures nominate a narrow, basic pocket on Rae1 engaged by the ORF6 C-terminus, supporting peptide mimetics or small-molecule PPI disruptors that compete for this site and ITC shows nM and sub-μM affinity for ORF6 C-terminus peptides [28,153,154]. In addition, host-pathway modulation (indirect) is also being implemented. The exportin 1 (XPO1) inhibitor Selinexor (Figure 8) was explored clinically in COVID-19 and partially rescued ORF6-induced phenotypes in cell systems in a dose-dependent manner, but it was not ORF6-selective and showed dose-limiting toxicities (DLTs) [155]. Other opportunities include biologics. Based on modelling and cell assays, IFN-γ has been proposed to bind the ORF6 C-terminus and displace Rae1, suggesting a blueprint for peptide and peptidomimetic decoys. IFN-γ itself is not typically used in COVID-19, but these data inform design [156].

#### 3.2.4. PK/PD and Safety

Any ORF6-targeted agent must reach the nuclear pore and ER–Golgi interface in the airway epithelium and avoid perturbing host nucleocytoplasmic transport. Preclinical rescue of ORF6 phenotypes by Selinexor supports the concept but clinical signals have been mixed and on-target toxicities of broad transport inhibition remain a constraint [155].

#### 3.2.5. Development Status and Resistance

ORF6 is a small accessory protein whose C-terminal region is functionally important for binding the Nup98–Rae1 complex and antagonising IFN signalling. Deletions and truncations have been observed in patients and clusters, generally correlating with attenuated phenotypic expression. Thus, resistance via loss-of-function would likely reduce virulence; a favourable trade-off for adjunctive therapeutics [157].

#### 3.2.6. Challenges and Opportunities

As a drug target, ORF6 presents several significant challenges. First, it acts through a PPI centred on the relatively shallow, basic Rae1-binding groove, which is inherently more difficult to target than a well-defined catalytic pocket. Second, the magnitude of its IFN-antagonist phenotype can vary substantially depending on the assay context, including cell type and expression level, complicating the interpretation of on-target activity. Third, because ORF6 acts by perturbing the host nuclear transport machinery, there is a potential risk of on-target toxicity if host trafficking is disrupted too broadly.

Despite these constraints, the target offers several clear opportunities. Structure-guided design may enable the development of peptide, peptidomimetic, or small-molecule disruptors of the ORF6–Rae1 interface. In parallel, poly-assay validation funnels integrating STAT1 nuclear import, mRNA export, and direct ORF6–Rae1 biophysical measurements should help reduce artefactual signals and strengthen confidence in target engagement. Most importantly, ORF6 antagonists are best positioned as adjuncts to direct-acting antivirals. By restoring IFN responsiveness and limiting immune-pathway dysregulation, they may improve overall therapeutic performance through a mechanism distinct from direct inhibition of viral proteases or polymerases [28,153,158].

### 3.3. ORF8: Immune Modulator and Evasion Protein

#### 3.3.1. Biology and Rationale

**Figure 9:**
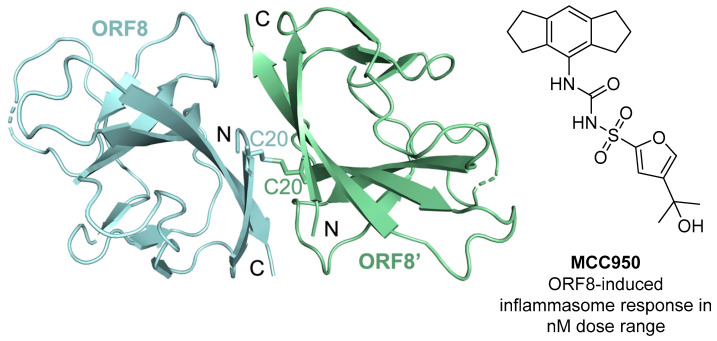
(**Left**): Crystal structure of the SARS-CoV-2 ORF8 dimer (PDB ID 7JTL). The two monomers are shown in cyan and green, and Cys20 from each monomer, forming the intermolecular disulfide bond, is shown as sticks. (**Right**): MCC950 [167,171], a selective NLRP3 inflammasome inhibitor used as a host-directed countermeasure to ORF8-driven IL-1β signalling.

ORF8 is a 121-amino-acid accessory glycoprotein with an immunoglobulin (Ig)-like fold that forms disulfide-linked dimers (Figure 9). It is capable of extracellular secretion, although the extent of secretion appears to be context- and variant-dependent [160,161,162]. ORF8 should be distinguished from the ORF8a/ORF8b arrangement described for SARS-CoV: in SARS-CoV, a 29-nucleotide deletion split the ancestral ORF8 into ORF8a and ORF8b, whereas SARS-CoV-2 encodes a single intact ORF8 protein [163]. Functionally, ORF8 downregulates surface MHC-I, thereby promoting escape from cytotoxic T-cell surveillance, in part by routing MHC-I toward lysosomal- and autophagy-linked degradation [29,164]. ORF8 has also been reported to act as a secreted pro-inflammatory factor, inducing cytokine responses and inflammasome-linked signalling in human monocytes. Several studies further propose IL-17-like signalling through IL-17 receptors (e.g., IL-17 receptor A; IL-17RA), although the precise downstream inflammatory mechanism remains under active debate [165,166,167]. Clinically, natural loss-of-function variants affecting the ORF8 region, most notably the Δ382 deletion, have been associated with milder disease, supporting the view that this axis contributes to virulence and may represent a therapeutically relevant target for attenuating disease severity [30,168].

#### 3.3.2. Assays and Structural Biology

Multiple crystal structures define ORF8 Ig-like dimers and interfaces suitable for ligand design. Structural comparisons with bat coronavirus ORF8 highlight the variability of surface loops that may underlie this functional divergence. Assay systems include flow cytometry for MHC-I surface levels in ORF8-expressing and infected cells. Colocalization with microtubule-associated protein 1A/1B-light chain 3 (LC3) and lysosomal markers to read out autophagy-dependent MHC-I degradation, and monocyte assays for inflammasome activation (IL-1β processing/ASC specks) and NF-κB/IL-17–axis readouts. Secreted ORF8 can be quantified in supernatants or plasma and used to stimulate primary monocytes [160,161,164,167,169].

#### 3.3.3. Chemical Matter

Direct small-molecule inhibitors of ORF8 have not yet been established. Current tractable strategies are largely biological- or pathway-targeted. On the one hand, neutralising antibodies against ORF8 reduce the binding of ORF8 to myeloid receptors and dampen its cytokine-inducing activity in cell systems, nominating antibody or decoy approaches to neutralise secreted ORF8 [170]. On the other hand, given ORF8’s NLRP3-dependent activation of monocytes, small-molecule NLRP3 inhibitors such as MCC950-class (Figure 9) are a rational adjunct to blunt ORF8-driven inflammation, which is a concept supported by mechanistic human-cell data [167,171]. Finally, studies reporting IL-17 mimicry show that blocking IL-17RA can reduce ORF8-induced inflammation in preclinical models, supporting anti-IL-17/IL-17RA biologics or peptide mimetics as route(s) to neutralise the virokine-like activity [165,172]. In silico reports of polyphenols or small molecules docking to ORF8 (e.g., at the dimer interface) exist, but experimental confirmation and potency remain limited; these should be treated as hypothesis-generating rather than validated leads [173].

#### 3.3.4. PK/PD and Safety

Because ORF8 is secreted, systemic or local (e.g., inhaled) biologics that neutralise extracellular ORF8 could achieve target engagement in the airway without entering cells. For pathway-directed approaches, NLRP3 blockade offers a downstream PD readout (IL-1β/IL-6 reduction); however, it requires careful safety profiling in virally infected hosts. Any small-molecule aimed at intracellular ORF8 mechanisms (e.g., MHC-I routing) would need to reach the endoplasmic reticulum (ER) and lysosomal compartments and avoid broad interference with antigen presentation [167].

#### 3.3.5. Development Status

As of 2026, no selective ORF8 inhibitors have been reported. Nevertheless, natural loss-of-function variants affecting the ORF8 region, including the Δ382 deletion, provide human genetic evidence that disruption of this virulence axis can be associated with attenuated disease, while accumulating structural and mechanistic data have defined assayable ORF8-linked phenotypes, notably MHC-I downregulation and monocyte and inflammasome activation, that can now support discovery efforts [29,30,162,174].

#### 3.3.6. Challenges and Opportunities

ORF8 presents several challenges as a therapeutic target. Its sequence varies across lineages, which may limit the durability of any intervention strategy. In addition, ORF8 is pleiotropic, with both intracellular effects on MHC-I modulation and extracellular cytokine-like activity, making it difficult to define a single dominant pharmacological readout. Its subcellular distribution also complicates targeting, as relevant functions may arise in the secreted form as well as within ER- and lysosome-associated compartments.

Despite these constraints, the target offers distinct opportunities. Its secreted component creates scope for neutralising antibodies or decoy-based approaches, while downstream pathway modulation, including blockade of NLRP3 or IL-17RA signalling, may help counter ORF8-driven inflammation. Simultaneously, structure-defined dimer interfaces provide a potential foundation for future small-molecule discoveries. Notably, this target may also offer a favourable resistance trade-off, since loss-of-function escape would be expected to attenuate virulence rather than enhance it [161,165,167].

### 3.4. ORF9b: Mitochondrial Antagonist of Interferon

#### 3.4.1. Biology and Rationale

ORF9b should be distinguished from the shorter overlapping N-frame product ORF9c. Unlike ORF9b, ORF9c remains much less well validated experimentally and is not supported by comparable structural or mechanistic evidence, so it is not discussed further here [131,175]. ORF9b (97 amino acids) is an accessory protein encoded by an alternative reading frame within the N gene. It localises to mitochondria and binds the import receptor TOM70, thereby disrupting the role of TOM70 as a scaffold for chaperone-delivered TBK1/IRF3 and dampening MAVS-dependent type I IFN signalling. Structural and cellular studies show that ORF9b engages a hydrophobic pocket on TOM70’s C-terminal domain, sterically/allosterically hindering heat shock protein 90 (Hsp90)–TOM70 complex formation and blunting IFN induction [176]. ORF9b also exhibits a phosphorylation switch at Ser50/Ser53: phosphomimetic changes weaken TOM70 binding and lessen IFN antagonism, suggesting host kinases can modulate ORF9b activity [177]. Together, these data position ORF9b as a virulence factor that subverts mitochondrial antiviral signalling and a plausible target to restore early innate responses [178].

#### 3.4.2. Assays and Structural Biology

**Figure 10:**
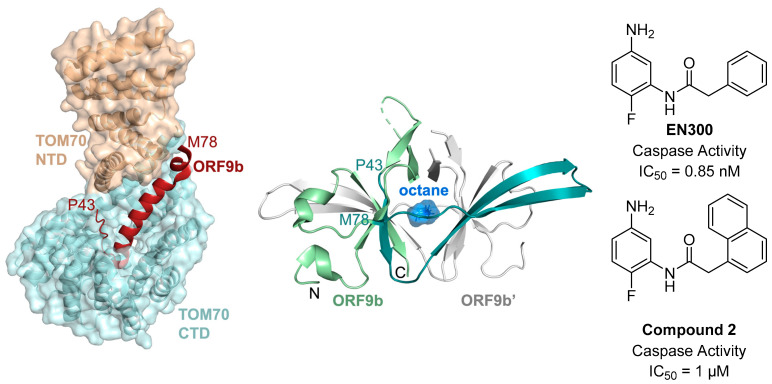
(**Left**): Structure of TOM70 in complex with the P43–M78 region of SARS-CoV-2 ORF9b. TOM70 is shown in beige and grey, and the ORF9b P43–M78 region is shown in red (PDB ID 7DHG). The N-terminal (NTD) N109-E248 and C-terminal (CTD) P249-K600 domains of TOM70 are shown in beige and cyan, respectively. (**Centre**): Crystal structure of the SARS-CoV-2 ORF9b dimer bound to lipid octane (PDB ID 9N55). The two ORF9b monomers are shown in green and grey, with the P43–M78 region in one monomer highlighted in teal. Lipid octane is shown in blue. (**Right**): Representative small-molecule chemotypes, EN300 and Compound 2, proposed to target the ORF9b dimer interface [183].

Cryo-EM and X-ray structures capture the central P43–M78 region of ORF9b bound to TOM70 (e.g., PDB ID 7DHG). In this complex, ORF9b binds as a monomeric α-helical segment within the TOM70 pocket, thereby rationalising how ORF9b occupancy can interfere with Hsp90-EEVD (Glu-Glu-Val-Asp) peptide binding [176,179,180]. This TOM70-bound state contrasts with isolated or lipid-bound ORF9b, which forms a β-sheet-rich homodimer with a lipid-binding tunnel (Figure 10). Lipid binding stabilises the ORF9b dimer and influences the monomer–dimer equilibrium, indicating that TOM70 engagement is coupled to a substantial conformational rearrangement of ORF9b. Functional readouts include IFN-β promoter reporter assays, co-immunoprecipitation, ITC for ORF9b–TOM70 binding, and peptide-competition assays measuring disruption of Hsp90–TOM70 interactions in the presence of ORF9b [181]. Recent biophysical work quantifies how ORF9b dimerisation, lipid binding, and TOM70 recognition are coupled, informing mechanism-aware screening [181].

#### 3.4.3. Chemical Matter

Drug-discovery proposals therefore focus on two tractable mechanisms: Disrupting the ORF9b–TOM70 PPI (guided by 7DHG and mutational hot-spots such as ORF9b S53/TOM70 E477) [177] and allosterically biasing ORF9b’s monomer–dimer–lipid equilibria to disfavour the TOM70-binding conformation [182]. Additionally, in silico and peptide-based efforts have begun: antiviral peptides designed to dock ORF9b’s pocket (and conceptually compete with TOM70) show μM-range cellular signals in preliminary studies, while a recent study described small molecules that inhibit ORF9b homodimerization as a surrogate for function (e.g., EN300 and Compound 2, Figure 10) [183]. These constitute starting points rather than drug-like leads and still require orthogonal, TOM70-centric cellular validation (e.g., restoration of IFN signalling and rescue of Hsp90–TOM70 binding).

#### 3.4.4. PK/PD and Safety

Because ORF9b acts at the outer mitochondrial membrane, candidates must have sufficient cell permeability and partition into ER/mitochondrial membranes without broadly perturbing mitochondrial import. On-target PD should restore early IFN responses (e.g., IFN-β reporter rescue; increased TBK1 phosphorylation and IRF3 nuclear translocation), whereas off-target risks include interference with TOM70’s essential import functions and Hsp90 client trafficking. Selectivity windows will likely require PPI-surface specificity rather than generic hydrophobic binders [177,184]. Timing is critical: the intended clinical use would be early in infection to re-enable innate control.

#### 3.4.5. Development Status

To date, no drug-like, advanced small-molecule ORF9b antagonists have been reported, although proof-of-concept chemical matter has begun to emerge. The most advanced work remains the structural and biophysical characterisation of ORF9b–TOM70 and early computational peptide concepts. There are no small-molecule inhibitors with demonstrated on-target rescue of IFN signalling in vivo. The growing structural and mechanistic base (PDB ID 7DHG), mapping of phosphorylation control, and quantitative conformational models) provides a realistic springboard for mechanism-guided PPI inhibitor discovery [177,181].

#### 3.4.6. Resistance, Challenges and Opportunities

ORF9b is relatively conserved across sarbecoviruses and embeds in a host-protein interface that may be mutationally constrained by fitness (mutations that weaken TOM70 binding would be expected to reduce IFN antagonism). Phospho-regulatory sites (e.g., Ser53) also suggest host-driven attenuation routes. Nonetheless, resistance via surface substitutions at the TOM70-contact patch is possible and should be monitored with deep mutational scanning once bona fide inhibitors exist. Given the accessory role of ORF9b, any escape could carry a virulence cost rather than a replication advantage [177].

### 3.5. ORF10: Cullin E3 Ligase Hijacker

#### 3.5.1. Biology and Rationale

**Figure 11:**
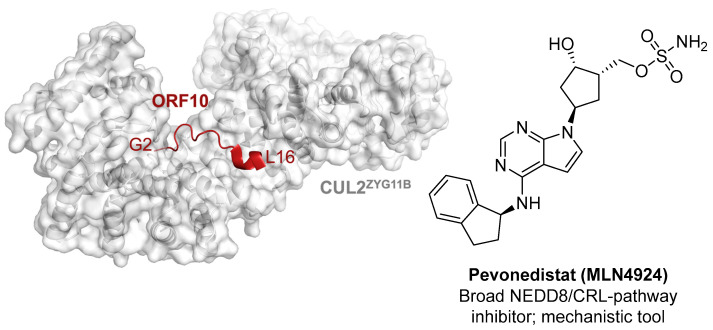
(**Left**): Cryo-EM structure of the CUL2^ZYG11B^ ligase, shown in grey, in complex with SARS-CoV-2 ORF10, shown in red (PDB ID 9BIE). The bound Elongin B/C complex is not included in the figure. (**Right**): Pevonedistat (MLN4924), an inhibitor of the NEDD8-activating enzyme that functionally inactivates cullin–RING ligases and blocks ORF10-driven hijacking of CRL2^ZYG11B^ [190].

ORF10 is an unusually small SARS-CoV-2 accessory protein (38 amino acids) encoded within the N locus (Figure 11). It is dispensable for replication in vitro and in vivo challenge models, indicating a non-essential role for basic replication, but it can modulate host pathways that influence disease biology [31]. Two host-hijacking mechanisms are supported by these findings. First, ORF10 engages the cullin-RING E3 ubiquitin ligase 2 containing zyg-11 family member B (CRL2^ZYG11B^) ubiquitin ligase via an N-terminal glycine degron. Furthermore, a recent co-crystal structure at 2.9 Å of CUL2^ZYG11B^ with the ORF10 N-terminal heptapeptide (PDB ID 7YC2) confirms that ORF10 mimics a Gly/N-degron, with G1 and Y2 anchoring recognition. Mutational disruption (e.g., G1P and Y2A) weakens ZYG11B binding and blunts intraflagellar transport protein 46 (IFT46) degradation in cells [185]. Functionally, ORF10 enhances CRL2^ZYG11B^ activity to trigger proteasomal degradation of ciliary protein IFT46, leading to loss of airway motile cilia and impaired mucociliary clearance in human nasal epithelial cultures and hACE2 mouse airways [186,187]. Second, ORF10 can induce mitophagy-mediated MAVS degradation through interaction with the mitophagy receptor NIX, also known as BCL2-interacting protein 3-like (BNIP3L), suppressing RIG-I/MAVS signalling and type I IFN induction [188]. While one study concluded the ORF10–CRL2^ZYG11B^ axis is not required for SARS-CoV-2 replication in standard cell culture, these pathways clarify how ORF10 may tune pathogenesis (cilia integrity and IFN antagonism) rather than core RNA replication [32].

#### 3.5.2. Assays and Structural Biology

Structural tools now include the ZYG11B–ORF10 peptide complex (PDB ID 7XV7), enabling structure-guided design of interface disruptors. The literature uses several assay classes to interrogate this axis [185,186,188]. These include biochemical and biophysical binding assays for the ZYG11B–ORF10 interaction, such as degron-competition studies by fluorescence polarization and ITC, alongside proteomic readouts of CRL2^ZYG11B^ activity. Cell-based assays include degradation reporters and pharmacological rescue with proteasome inhibitors (e.g., MG132) or neddylation inhibitors (e.g., MLN4924/pevonedistat) to confirm cullin-RING ligase dependence. Additional models include airway epithelial systems that quantify ciliary protein abundance and motile-cilia coverage, together with IFN-β promoter or interferon-stimulated gene reporter assays to assess reversal of ORF10-mediated innate-immune suppression through the NIX–MAVS pathway.

#### 3.5.3. Chemical Matter

Tool compounds support tractability at the pathway level: MLN4924, also known as Pevonedistat (Figure 11), blocks neural precursor cell expressed, developmentally downregulated 8 (NEDD8) activation and thereby broadly disables cullin–RING ligase (CRL) E3 ubiquitin ligases. In cell systems, MLN4924 blocks CRL2^ZYG11B^-dependent substrate degradation, providing a useful mechanistic control, although its broad activity and toxicity make it unsuitable as a direct antiviral therapy. The ZYG11B–ORF10 complex structure recommends peptide and peptidomimetic degron decoys or small molecules targeting the ZYG11B substrate pocket as rational starting points [185,186,187].

Reports that Amantadine (*vide supra*) blocks ion-channel activity ascribed to ORF10 in *Xenopus oocytes* are preclinical and are debated. The E protein is an accepted viroporin target of Amantadine, whereas assigning bona fide ion-channel function to ORF10 requires stronger criteria. Accordingly, any “ORF10 channel blocker” claim should be considered provisional [87,189]. Finally, for the mitophagy route, the ORF10–NIX interaction nominates interface-blocking peptides or small molecules as a concept, but no selective molecules have been published to date [188].

#### 3.5.4. PK/PD and Safety

Given the host-complex targets of ORF10, on-target liabilities are a concern for pathway-level inhibitors. For example, MLN4924 inhibits NEDD8 activation and thereby broadly suppresses cullin-RING ligase activity, rather than selectively blocking the ORF10–CRL2^ZYG11B^ interaction. Such broad inhibition would be expected to disrupt many ubiquitination pathways and is therefore more suitable as a mechanistic tool than as a selective antiviral strategy. Interface-targeted agents (ZYG11B pocket binders or NIX-interface blockers) could, in principle, achieve selectivity, but would require careful off-target and immune-activation window profiling, as amplifying IFN signalling late in disease can worsen inflammation [186].

#### 3.5.5. Development Status

As of 2026, no validated selective small-molecule ORF10 inhibitors have been reported. Structural and mechanistic advances (ZYG11B–ORF10 complex, CRL2^ZYG11B^, and NIX-dependent phenotypes) establish tractable hypotheses and screening strategies for PPI-disruptors and degron-competitive ligands, but programs remain in early stages [187].

#### 3.5.6. Resistance

Because ORF10 is non-essential for replication in standard systems and is sometimes truncated in clinical isolates, resistance via loss or mutation of ORF10 would likely carry a limited fitness cost for the virus. That argues ORF10-targeted agents would be best positioned as adjuncts to replication-directed DAAs (e.g., M^pro^ or RdRp inhibitors) to reduce pathogenesis (cilia loss, IFN antagonism) rather than as monotherapy [31].

#### 3.5.7. Challenges and Opportunities

Key challenges include the very small size of ORF10 (classical pocket scarcity) and the host-targeted nature of its mechanisms. Opportunities stem from structure-enabled intervention at the ZYG11B substrate pocket, airway-relevant phenotypic assays (cilia preservation in human nasal epithelial cells) that provide translational readouts, and potential immunologic synergy. Additionally, blocking ORF10 could preserve cilia function and restore MAVS-dependent IFN signalling, complementing DAAs early in infection [186,188].

### 3.6. Other Accessory Proteins

Among the accessory proteins not examined in detail, ORF7a is the most immediately relevant. Its reported antagonism of BST-2 (tetherin), together with broader roles in host pathway manipulation, makes it a plausible adjunctive target, although direct chemical validation remains very limited [191]. Beyond ORF7a, several additional accessory products are biologically interesting but are not discussed here in depth because they currently lack the structural, mechanistic, or chemical maturity needed for prioritised small molecule evaluation. ORF7b remains comparatively under-characterised, while ORF3b, ORF3c, ORF3d, and ORF9c have been proposed or implicated in immune modulation, interferon antagonism, or lineage-specific phenotypes [20,175,192,193]. For most of these proteins, however, target validation remains incomplete, structural information is limited or absent, and experimentally supported small molecule chemical matter is scarce. They are therefore best regarded at present as hypothesis-generating or lower-priority adjunctive opportunities rather than near-term medicinal chemistry programmes.

## 4. Discussion

### 4.1. Translational Hierarchy of Structural and Accessory Protein Targets

The structural and accessory proteomes of SARS-CoV-2 define a complementary antiviral landscape whose value lies not in replacing clinically validated non-structural enzyme targets but in expanding the mechanistic diversity of therapeutic interventions. Whereas the first generation of successful DAAs was built around the inhibition of viral proteolysis and RNA synthesis, the proteins discussed in this review govern entry, fusion, assembly, egress, and immune-pathway manipulation, processes that are upstream, downstream, or orthogonal to the replication–transcription complex [10,11,12]. Consequently, the central translational question is not whether these targets can substitute for the non-structural proteome, but which of them are sufficiently tractable, validated, and biologically important to justify development as direct or adjunctive antiviral targets.

From this perspective, the current target hierarchy is not evenly distributed. At present, the strongest disclosed direct small-molecule target outside the non-structural proteome is the M protein. Historically regarded as a difficult membrane scaffold, M has emerged as a credible antiviral target through the identification of independent chemotypes, such as JNJ-9676 and CIM-834, supported by direct binding, mapped resistance, cellular antiviral activity in physiologically relevant systems, and oral efficacy in animal infection models [106,107]. These findings reposition M from a conceptual assembly target to a mechanistically validated antiviral node and provide rare evidence that a coronavirus structural protein can support direct-acting small-molecule discovery at a level approaching genuine preclinical maturity. In contrast, N remains attractive but at an earlier stage, with promising allosteric and interface-focused chemical matter but still limited progression beyond tool compounds and assay-development chemistry [98,99,108,109,110,111,112,113,114,115,116]. E and ORF3a retain clear biological interest as viroporin-like proteins implicated in membrane remodelling, egress, and inflammatory signalling. Nevertheless, the gap between mechanistic plausibility and robust on-target small-molecule validation remains wider than that for M [194,195]. The S protein remains biologically central, but for small-molecule discovery, it occupies a more selective and difficult niche [196,197]. Its high sequence variability, glycan shielding, and dominance of biologic modalities make it a less robust foundation for durable small-molecule antiviral design than the strongest assembly-linked targets.

### 4.2. Druggability and Structural Biology of Non-Canonical Antiviral Targets

From a medicinal chemistry perspective, the structural and accessory proteomes impose a distinct set of constraints compared with catalytic non-structural enzymes. Many of these proteins are membrane-associated, oligomeric, conformationally plastic, or dominated by protein–protein and protein–RNA interfaces rather than well-defined catalytic pockets [18]. Consequently, the most useful ligands in this space often emerge not from classical substrate-mimetic logic but from conformational locking, allosteric modulation, interface disruption, or phenotypically guided discovery.

Recent structural biology has substantially changed this landscape. Cryo-EM and NMR studies have defined conformational states and ligandable regions in several previously challenging targets, including M, E, ORF3a, ORF6, ORF9b, and ORF10. These advances are particularly important for membrane and host-interface proteins, where druggability may depend on transient pockets, oligomeric interfaces, or conformational states that are not obvious from sequence analysis alone. The M protein provides the clearest example: ligand-induced pockets and conformation-locking mechanisms have converted an apparently difficult membrane scaffold into a credible small-molecule assembly target [106,107]. For N, structural information on the NTD, CTD, RNA-binding surfaces, and allosteric pockets has enabled ligand discovery, although the intrinsically disordered linker region, multivalent RNA binding, and LLPS behaviour continue to complicate chemical optimisation.

Across the organic small molecules discussed in this review, the most informative structure–target relationships are target-class-specific rather than universal. For S, durable chemical matter is likely to require engagement of conserved allosteric or fusion-associated sites rather than exposed and highly variable RBD/NTD surfaces. For M, the clearest emerging relationship is between compact, membrane-compatible chemotypes and ligand-induced conformational locking at the transmembrane dimer interface, as illustrated by JNJ-9676 and CIM-834. For E and ORF3a, several reported modulators contain cationic, amphiphilic or membrane-partitioning features compatible with viroporin or membrane-associated activity, but these same properties increase the risk of nonspecific membrane perturbation and ion-channel promiscuity. For N, early active chemotypes are often anionic, polyphenolic, polyanionic or hydrogen-bond-rich scaffolds that engage basic RNA-binding or allosteric surfaces, but these features also create permeability, selectivity and PK liabilities. For host-interface accessory proteins such as ORF6, ORF8, ORF9b and ORF10, the relevant chemical space is less mature and may favour interface-directed or allosteric chemotypes rather than classical active-site inhibitors. Thus, chemical structure–target relationships can already guide prioritisation, but most remain preliminary and require systematic SAR, orthogonal target engagement and developability optimisation before they can support true candidate selection.

### 4.3. Screening Platforms and Target-Engagement Validation

Because many structural and accessory protein targets lack canonical enzymatic activity, screening strategies must be adapted to the biological function of each protein. Classical biochemical assays are useful where defined binding or displacement events can be measured, for example RBD–ACE2 binding, N–RNA binding, NTD/CTD ligand binding, or ORF6–Rae1/Nup98 and ORF9b–TOM70 interactions. However, these assays are rarely sufficient on their own. For membrane and assembly-linked targets, high-content phenotypic screening, VLP systems, pseudovirus assays, authentic-virus assays, ion-flux assays, LLPS readouts, co-immunoprecipitation, and target-engagement assays all provide complementary information. In practice, discovery campaigns in this space are likely to require a combination of high-throughput screening, structure-guided virtual screening, fragment-based approaches, and phenotypic assays, depending on whether the target offers a defined binding pocket, a measurable protein–protein or protein–RNA interaction, or primarily a functional cellular phenotype.

The key requirement is orthogonal validation. For membrane proteins such as M, E, and ORF3a, compelling evidence should ideally integrate direct binding or structural localisation of the ligand, a functional assay linked to the relevant stage of the viral life cycle, and a cellular phenotype that cannot be readily explained by nonspecific membrane perturbation. For N and other assembly-linked factors, biochemical RNA-displacement assays, LLPS microscopy, and VLP-based systems are informative only when coupled with more direct measures of target engagement and infectious output. Similarly, for host-interface accessory proteins such as ORF6, ORF8, ORF9b, and ORF10, screening platforms must distinguish true modulation of the viral protein–host pathway from broad perturbation of nuclear transport, inflammation, mitochondrial function, ubiquitination, or innate immune signalling. In practice, the evidentiary threshold should therefore be higher, not lower, for non-canonical antiviral targets.

### 4.4. Mutation, Conservation, and Combination Therapy

A major strategic advantage of expanding beyond the non-structural enzyme targets is the possibility of combining mechanistically orthogonal interventions. An assembly inhibitor targeting M, an entry-directed modulator acting on S, or a virulence-focused adjunct affecting ORF3a, ORF6, or ORF9b is expected to impose different evolutionary and functional pressures on the virus than an M^pro^ or RdRp inhibitor [198]. Mechanistic orthogonality is valuable because it can reduce dependence on a single class of replication-directed agents, increase the barrier to escape, and improve biological coverage across different phases of infection.

However, not all targets are equally suited for resistance management or broad-spectrum preparedness. M and, to a lesser extent, N appear functionally constrained and relatively conserved, making them stronger candidates for variant-resilient or broader sarbecovirus-oriented development than S. In contrast, S is highly exposed and genetically labile, especially in the RBD and NTD, and therefore remains vulnerable to immune and therapeutic escape [42,199]. Accessory proteins may confer important in vivo vulnerabilities, but their sequence variability, context-dependent phenotypes, and frequent roles in virulence rather than core replication make them less reliable as stand-alone preparedness anchors. These features support a development logic in which the most mature assembly-linked structural targets are prioritised for direct antiviral optimisation, while host-interface accessory proteins are advanced more selectively as adjunctive or pathology-modifying components of combination regimens.

### 4.5. Challenges in Developing Small-Molecule Inhibitors

The development of small-molecule inhibitors against structural and accessory proteins faces several target-class-specific challenges. Membrane proteins such as M, E, and ORF3a require assays that preserve relevant oligomeric states, lipid environments, and subcellular localisation. Apparent activity against these targets can be confounded by nonspecific membrane perturbation, ion-channel promiscuity, or disruption of organelle homeostasis. Intrinsically disordered or phase-separating proteins such as N present a different problem: compounds may perturb condensates or RNA interactions without producing selective or drug-like target engagement [200]. Similarly, accessory proteins that act through host complexes raise a narrow therapeutic window, because pathway-level inhibition can disrupt essential host processes.

Developability also remains important. Potency alone may be misleading if adequate exposure is not achieved at the relevant site of action, such as the airway epithelium, ERGIC/Golgi compartment, endolysosomal system, mitochondrial interface, or virion assembly site. The encouraging performance of M-targeting compounds in primary airway systems and oral animal models illustrates the type of translational evidence needed for this class: not only biochemical or structural plausibility, but a clear pharmacodynamic link between target engagement, reduced infectious virus, and practical deployability [106,107].

### 4.6. Strategic Prioritisation

Overall, the most credible path forward is not to treat all structural and accessory proteins as equivalent drug-discovery opportunities. Instead, future programmes should prioritise targets with convergent evidence across structure, chemistry, cellular activity, resistance mapping, and in vivo pharmacodynamics. Such programmes should combine structure-enabled design, robust screening platforms, orthogonal target-engagement validation, resistance-aware development, and delivery-conscious pharmacology. The comparative framework summarised in Table 1 highlights the current balance of biological rationale, chemical tractability, translational maturity, and likely therapeutic role across the principal structural and accessory protein targets discussed in this review.

## 5. Conclusions

The structural and accessory proteomes of SARS-CoV-2 define a complementary antiviral landscape to the enzyme-rich non-structural proteome. Their principal value lies not in replacing the clinically validated benchmark targets of the replication–transcription complex, but in broadening the mechanistic scope of antiviral intervention through orthogonal disruption of entry, fusion, assembly, egress, and host-interface virulence functions. As the field has matured, it has become increasingly clear that these targets should not be viewed as uniform classes. Rather, they span a continuum from genuinely emerging direct-acting opportunities to more exploratory or adjunctive strategies, whose therapeutic value may depend on combination use and careful biological context.

At present, the clearest direct small-molecule opportunity in this landscape lies in assembly-linked structural targets, particularly M, whereas N and E remain earlier-stage opportunities and S is best pursued selectively. By contrast, accessory proteins are more credibly positioned as adjunctive or virulence-modifying targets than as stand-alone antiviral anchors. Future progress will therefore depend on disciplined prioritisation: advancing the strongest structural programmes first, and pursuing accessory-protein strategies only where rigorous target-engagement data in physiologically relevant systems support a clear translational rationale. Under this framework, structural and accessory proteins are unlikely to displace the best-established coronavirus antiviral targets, but they can broaden the therapeutic toolkit by adding mechanistically distinct and potentially resistance-complementary intervention points.

## Figures and Tables

**Table 1:** Strategic prioritisation of SARS-CoV-2 structural and accessory protein targets for small-molecule antiviral drug development.

Target	Principal Biological Role	Most Plausible Small-Molecule Strategy	Strategic Positioning	Current Evidence Maturity	Main Translational Challenge	Suggested Development Priority
**Membrane (M)**	Virion assembly, envelope organisation, budding/egress	Conformation-locking or oligomerisation-disrupting inhibitors	Direct-acting structural target	Highest among these targets; direct binding, resistance mapping, cellular activity, and in vivo proof-of-concept support genuine preclinical tractability	Membrane-protein assay complexity; maintaining selectivity and developability in a hydrophobic target environment	Highest priority
**Nucleocapsid (N)**	Genome encapsidation, ribonucleoprotein organisation, assembly-linked RNA interactions	Allosteric modulation, RNA-interface disruption, condensate/assembly perturbation	Direct-acting but earlier-stage structural target	Moderate; biologically compelling and relatively conserved, but still dominated by tool compounds and assay-development chemistry rather than mature lead series	Proving on-target activity in complex RNA-/phase-separation-driven systems; converting tool chemistry into true leads	High priority (second wave)
**Envelope (E)**	Membrane remodelling, assembly, egress, viroporin-linked functions	Channel modulation or viroporin interference	Potential direct-acting structural target	Early to moderate; strong biological rationale but less convincing direct small-molecule validation than M	Distinguishing true on-target viroporin inhibition from nonspecific membrane effects	Medium priority
**Spike (S)**	Receptor engagement, membrane fusion, viral entry	Entry/fusion modulation (direct small molecules only)	Selective direct-acting opportunity	Mixed; biologically central, but small-molecule programmes remain less mature and are overshadowed by biologics	High sequence variability, glycan shielding, conformational plasticity, and weaker durability for small-molecule design	Medium priority, highly selective
**ORF3a**	Membrane trafficking, egress-linked functions, inflammasome/stress signalling	Viroporin-like modulation or trafficking-linked functional disruption	Borderline direct/adjunctive target	Early; compelling biology but a wider gap between mechanistic plausibility and validated on-target chemical matter	Complex membrane biology; risk of indirect phenotypes; need for stronger target-engagement evidence	Medium-to-lower priority
**ORF6**	Interferon antagonism via nuclear pore interference	Disruption of host-interface protein–protein interactions	Adjunctive/virulence-modifying target	Early; strong mechanistic rationale but highly context-dependent	Demonstrating selective, on-target modulation of host-interface signalling without broad host liabilities	Selective adjunctive priority
**ORF8**	Immune evasion, including effects on antigen presentation	Interface-disrupting or trafficking-modulating strategies	Adjunctive/virulence-modifying target	Early; biologically interesting but variable and context-sensitive	Sequence variability, uncertain durability, and the difficulty of linking target modulation to clear antiviral benefit	Selective adjunctive priority (lower priority)
**ORF9b**	Suppression of innate immune sensing through TOM70/MAVS-linked pathways	Host-interface protein–protein interaction disruption	Adjunctive/virulence-modifying target	Early; attractive as a host-interface target but not yet a strong stand-alone chemical programme	Strong dependence on physiologically relevant assay systems; limited direct chemical validation	Selective adjunctive priority (lower priority)
**ORF10**	Uncertain; proposed host-interface effects remain debated	Exploratory interface-disrupting strategies	Hypothesis-generating/highly speculative target	Very low; biological relevance and druggability remain uncertain	Target validity itself remains unresolved	Lowest priority
**Other accessory proteins (e.g., ORF7a/b, ORF3b/3c/3d, ORF9c)**	Host-interface modulation, interferon antagonism, virion egress, and lineage-specific virulence functions	Exploratory protein–protein interaction disruption or pathway-modulating adjunct strategies	Hypothesis-generating/lower-priority adjunctive targets	Very low to early; biologically relevant but generally lacking complete target validation, structural definition, and experimentally supported small-molecule chemical matter	Uncertain target validity, limited structural/mechanistic resolution, and minimal on-target chemical validation in physiologically relevant systems	Reserve for exploratory or follow-on programmes (lowest priority)

## Data Availability

Not applicable.

## Abbreviations

The following abbreviations are used in this manuscript:

ACE2	Angiotensin-converting enzyme 2
ALI	Air–liquid interface
ASC	Apoptosis-associated speck-like protein containing a caspase recruitment domain
BD(s)	β-sheet domain(s)
BID	Twice daily
BLI	Biolayer interferometry
BNIP3L	BCL2/adenovirus E1B 19 kDa protein-interacting protein 3-like (NIX)
BST-2	Bone marrow stromal cell antigen 2 (tetherin)
CD317	Cluster of differentiation 317
CD(s)	Cytosolic domain(s)
CNS	Central nervous system
Co-IP	Co-immunoprecipitation
CoV	Coronavirus
COVID-19	Coronavirus Disease 2019
CRL	Cullin–RING ligase
CRL2^ZYG11B^	Cullin–RING ligase 2 containing zyg-11 family member B
CUL2^ZYG11B^	Cullin 2–ZYG11B E3 ubiquitin ligase complex
Cryo-EM	Cryo-electron microscopy
CTD	C-terminal dimerisation/RNA-binding domain (of N protein)
DAA(s)	Direct-acting antiviral(s)
DLT(s)	Dose-limiting toxicity/toxicities
DOPE	1,2-dioleoyl-sn-glycero-3-phosphoethanolamine
E	Envelope (protein)
E3	E3 ubiquitin ligase
EC_50_	Half-maximal effective concentration
EC_90_	90% effective concentration
EEVD	Glu-Glu-Val-Asp peptide motif
ELISA	Enzyme-linked immunosorbent assay
EMSA	Electrophoretic mobility shift assay
ER	Endoplasmic reticulum
ERGIC	ER–Golgi intermediate compartment
HCoV	Human coronavirus
HE	Hemagglutinin-esterase
HIV-1	Human immunodeficiency virus type 1
HMA	Hexamethylene amiloride
HOPS	Homotypic fusion and vacuole protein sorting
Hsp90	Heat shock protein 90
IC_50_	Half-maximal inhibitory concentration
IDR	Intrinsically disordered region
IFN	Interferon
IFT46	Intraflagellar transport protein 46
Ig	Immunoglobulin
IL	Interleukin
IL-17RA	Interleukin-17 receptor A
IRF3	Interferon regulatory factor 3
ISG	Interferon-stimulated gene
ISGF3	Interferon-stimulated gene factor 3
ITC	Isothermal titration calorimetry
JM	Juxtamembrane
K18-hACE2	Keratin 18 human angiotensin-converting enzyme 2 transgenic model
K_d_	Equilibrium dissociation constant
LC3	Microtubule-associated protein 1A/1B-light chain 3
LKR	Ser/Arg-rich linker
LLPS	Liquid–liquid phase separation
M	Membrane (protein)
mAb(s)	Monoclonal antibody/antibodies
MAVS	Mitochondrial antiviral-signalling protein
MDA5	Melanoma differentiation-associated protein 5
MERS-CoV	Middle East Respiratory Syndrome Coronavirus
MHC-I	Major histocompatibility complex class I
MHV	Mouse hepatitis virus
MRC	Medical Research Council (UK)
M^pro^	Main protease (Nsp5)
mRNA	Messenger RNA
N	Nucleocapsid (protein)
NEDD8	Neural precursor cell expressed, developmentally downregulated 8
NF-kB	Nuclear factor kappa B
NIX	Mitochondrial outer-membrane receptor BNIP3L (see BNIP3L)
NLRP3	NOD-, LRR- and pyrin domain-containing protein 3
NMR	Nuclear magnetic resonance
Nsp(s)	Non-Structural Protein(s)
NTD	N-terminal RNA-binding domain (of N protein)
Nup98	Nucleoporin 98
ORF	Open reading frame
PALS1	Protein associated with Lin Seven 1
PDB	Protein Data Bank
PK/PD	Pharmacokinetics/pharmacodynamics
PPI	Protein–protein interaction
QD	Once daily
Rae1	RNA export 1
Rab7	Ras-related protein Rab-7a
RBD	Receptor-binding domain (of Spike)
RBP(s)	RNA-binding protein(s)
RdRp	RNA-dependent RNA polymerase (Nsp12)
RIG-I	Retinoic acid-inducible gene I
RNA	Ribonucleic acid
RNP	Ribonucleoprotein
S	Spike (glycoprotein)
SARS-CoV	Severe Acute Respiratory Syndrome Coronavirus (2002–2003)
SARS-CoV-2	Severe Acute Respiratory Syndrome Coronavirus 2
SCID	Severe combined immunodeficiency
SPR	Surface Plasmon Resonance
STAT	Signal transducer and activator of transcription (e.g., STAT1/STAT2)
TBK1	TANK-binding kinase 1
TM	Transmembrane
TMPRSS2	Transmembrane protease, serine 2
TOM70	Translocase of outer mitochondrial membrane 70
TRAF3	Tumour necrosis factor receptor-associated factor 3
VLP	Virus-like particle
Vpu	Viral protein U
VSV	Vesicular stomatitis virus
XPO1	Exportin 1
ZYG11B	Zyg-11 family member B
